# Budigalimab, an anti-PD-1 inhibitor, for people living with HIV-1: a randomized, placebo-controlled phase 1b study

**DOI:** 10.1038/s41591-025-03993-0

**Published:** 2025-10-15

**Authors:** Moti N. Ramgopal, Jacob P. Lalezari, Ana Gabriela Pires dos Santos, Preethi Krishnan, Tanaya R. Vaidya, Fei Zhou, Heide Betman, Patrick Dorr, Nael M. Mostafa, Maria L. Alcaide, Franco Felizarta, Jean-Pierre Routy

**Affiliations:** 1 Midway Immunology and Research Center, Fort Pierce, FL USA; 2 Quest Clinical Research, San Francisco, CA USA; 3 AbbVie Inc., North Chicago, IL USA; 4 University of Miami Miller School of Medicine, Miami, FL USA; 5 Office of Franco Felizarta, MD, Bakersfield, CA USA; 6 Research Institute of the McGill University Health Centre, Montreal, Quebec Canada; 7 Chronic Viral Illness Service and Division of Hematology, McGill University Health Centre, Montreal, Quebec Canada

**Keywords:** Drug development, Outcomes research, Immunotherapy, HIV infections

## Abstract

Chronic human immunodeficiency virus type 1 (HIV-1) disease results in immune exhaustion and dampened T cell responses, and programmed cell death 1 (PD-1) inhibitors offer a potential approach to enable viral control without antiretroviral therapy (ART) through reversal of these effects. Budigalimab is an investigational humanized anti–PD-1 monoclonal antibody. Multiple intravenous (IV) low doses of budigalimab (Stage 1: 2 mg *n* = 10, 10 mg *n* = 10, placebo *n* = 5, two doses every 4 weeks; Stage 2: 10 mg *n* = 11, placebo *n* = 5, four doses every 2 weeks (Q2W)) were assessed in people living with HIV (PLWH; *n* = 41) in a randomized, double-blind, multicenter, placebo-controlled phase 1 study with an analytical treatment interruption (ATI) to identify an efficacious regimen with a favorable safety profile in PLWH. The primary endpoints were safety, tolerability and pharmacokinetics. Demographics and baseline characteristics were balanced across treatment groups, except for sex, which was mostly male. All participants identified as cisgender. Budigalimab was well tolerated for up to 44 weeks, with 29 of 41 participants experiencing an adverse event (AE), including 2 participants who each experienced one grade 1 reversible immune-related AE (thyroiditis, hyperthyroidism). Three grade 3 AEs were reported by two participants and one serious AE by one participant; none were deemed related to treatment. IV budigalimab 10 mg Q2W resulted in a slight accumulation of drug in serum, with concentrations remaining above the estimated concentration required for near-complete (>95%) PD-1 receptor saturation on CD8^+^ T cells for ~10 weeks in peripheral blood. In an exploratory efficacy analysis of a 12-week ATI initiated with the first of four 10 mg Q2W doses, 6 of 11 participants experienced a delayed rebound with a relatively low viral peak and/or off-ART viral control (<200 copies ml^−1^) for ≥6 weeks during ATI, with 2 sustaining ART-free viral control to study end (204−252 days). The study achieved prespecified endpoints, supporting further evaluation of budigalimab in PLWH in a phase 2 study. ClinicalTrials.gov identifier: NCT04223804.

## Abstract (web summary)

A randomized, controlled phase 1b trial evaluated the safety, pharmacokinetics and impact on viral kinetics of budigalimab, a PD-1 inhibitor, during analytical treatment interruption **for HIV** and found that it was well tolerated and resulted in delayed rebound in a subset of participants in an exploratory efficacy analysis.

## Main

Human immunodeficiency virus type 1 (HIV-1) leads to a chronic condition that remains a major global health problem, with an estimated 40 million people worldwide living with HIV in 2023 (ref. ^1^). Antiretroviral therapy (ART) is effective in maintaining viral suppression to prevent the development of opportunistic infection and HIV-1-related death. However, ART is not curative, and persistent low-level inflammation remains^2–4^, as does the risk of comorbidities^5–7^ and long-term ART-related toxicities^8,9^ and challenges to maintaining optimal levels of adherence^10^, which is critical to avoid drug resistance. ART use is often affected by HIV-related stigma and associated mental health conditions, reduced self-efficacy or concerns of disclosure^11,12^. An HIV treatment strategy of finite duration that leads to sustained viral suppression without ART, even one requiring periodic retreatment, would fundamentally alter the current HIV treatment paradigm from one of lifelong daily treatment to one of prolonged periods of no treatment punctuated by intermittent therapy. This strategy could reduce the clinical effects of HIV infection and treatment and substantially improve the lives of people living with HIV (PLWH). Such a treatment strategy remains a research priority, including for the US National Institutes of Health and International AIDS Society^13,14^.

HIV-specific CD8^+^ T cell responses are key to controlling viral replication and establishing a setpoint viral load during early infection^15,16^. Over time, HIV and many other pathogens escape this immune control through the promotion of inhibitory intercellular interactions via immune checkpoint proteins, as observed in the upregulated expression of proteins such as programmed cell death 1 (PD-1) on both CD4^+^ and CD8^+^ T cells in untreated HIV infection^17–20^. The activity of PD-1 has been implicated in the CD8^+^ T cell exhaustion observed in chronic HIV infection^21–23^. PD-1 is upregulated on activated T cells and interacts with its ligands, PD-L1 or PD-L2, driving a dominant negative checkpoint signal that limits subsequent cellular activation through antigen receptors^24–26^. HIV-specific CD8^+^ T cells expressing high levels of PD-1 and other immune checkpoint receptors are characterized by reductions in cellular proliferation, cytokine production and cytotoxic effects^17,18,27,28^. Also, it has been shown that immune checkpoint markers including PD-1 are expressed on CD4^+^ T cells harboring intact proviruses and potentially play a role in conferring resistance to immune-mediated clearance of these cells^29^. PD-1 blockade in non-human primates infected with simian immunodeficiency virus has resulted in multiple effects, including enhancement of HIV-specific CD8^+^ T cell proliferative potential in the blood and gut and increased polyfunctional capacity; promotion of CD8^+^ T cell response against gut-resident pathogens leading to reduced gut epithelial damage, microbial translocation and immune activation; expansion of CXCR5^+^perforin^+^granzyme B^+^ effector CD8^+^ T cells, whose targets include lymphoid follicular cells, a reservoir for HIV; and reductions in setpoint viral load^30–33^. PD-1 inhibitors, therefore, offer a potential approach to enable durable viral control without ART through a reversal of T cell exhaustion and restoration of T cell function.

Budigalimab (ABBV-181) is an investigational humanized, recombinant immunoglobulin G1 (IgG1) L234A L235A monoclonal antibody (mAb) that effectively binds to cell surface-expressed PD-1 and blocks it from binding to PD-L1 and PD-L2 (ref. ^34^). Phase 1 data examining budigalimab in solid tumors showed that a dosing regimen of 250 mg intravenously (IV) every 2 weeks (Q2W) or 500 mg IV every 4 weeks (Q4W) can be administered with an acceptable safety profile in patients with cancer and demonstrates antitumor activity in tumor types known to be susceptible to PD-1-targeting agents^34,35^. Herein, we report on budigalimab as a potential treatment for HIV-1 infection. Study M19-939 (NCT04223804) was a phase 1b randomized, placebo-controlled double-blind study to investigate the safety, pharmacokinetics and pharmacodynamics of multiple doses of budigalimab in PLWH. The doses chosen for study in HIV treatment were derived from modeling using data from first-in-human studies of budigalimab^34^ and pharmacokinetic studies of another anti-PD-1 antibody with target-mediated drug disposition^36^ and were substantially lower and of shorter exposure duration than those used in oncology. The main objectives of the current study were to evaluate the safety and tolerability, specifically drug-related grade ≥3 adverse events (AEs), study drug-related AEs, immune-related AEs (IRAEs) and AEs corresponding to retroviral rebound syndrome and the pharmacokinetics of repeat dosing of budigalimab in PLWH while on suppressive ART and during analytical treatment interruption (ATI). Exploratory analyses included saturation of PD-1 receptor, biomarkers and viral load kinetics with repeat dosing of budigalimab during ATI.

## Results

### Patient disposition

**Extended Data Fig. 1:**
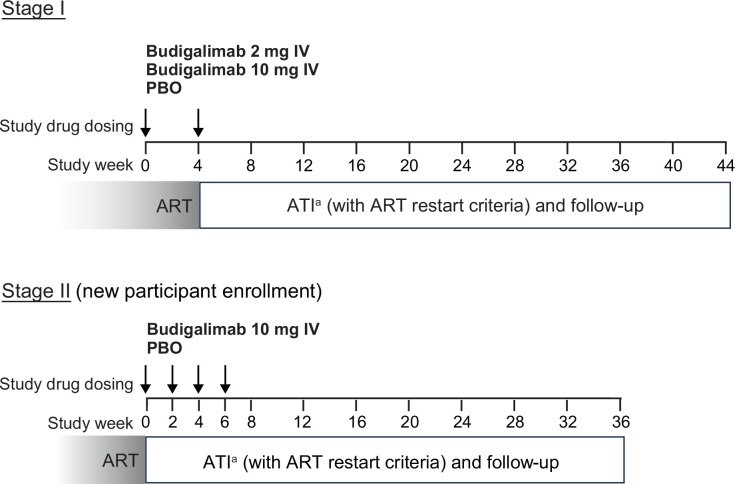
Study design. aATI planned for 12 weeks; ART-restart criteria were ≥1 of the following: ≥2 consecutive plasma HIV-1 RNA measures ≥1000 copies/mL for ≥4 weeks; ≥2 consecutive measures of >30% decline from baseline CD4 + T cell count or absolute CD4 + T cell count <350 cells/µL; HIV-associated symptoms, including symptoms of retroviral rebound syndrome; pregnancy; or participant or investigator request to reinitiate ART. ART, antiretroviral therapy; ATI, analytical treatment interruption; IV, intravenous; PBO, placebo.

The study used a two-stage design (Extended Data Fig. 1). In stage I, participants on suppressive ART received two IV doses of budigalimab 2 mg (*n* = 10) or 10 mg (*n* = 10) or placebo (*n* = 5) once every 4 weeks (Q4W×2). After receiving the second dose at week 4, participants initiated a closely monitored ATI. Following an assessment of safety, pharmacokinetic and pharmacodynamic data at stage I, a separate group of participants was enrolled in stage II and received four IV doses of budigalimab 10 mg (*n* = 11) or placebo (*n* = 5) once every 2 weeks (Q2W×4) while initiating an ATI on day 1.

**Fig. 1:**
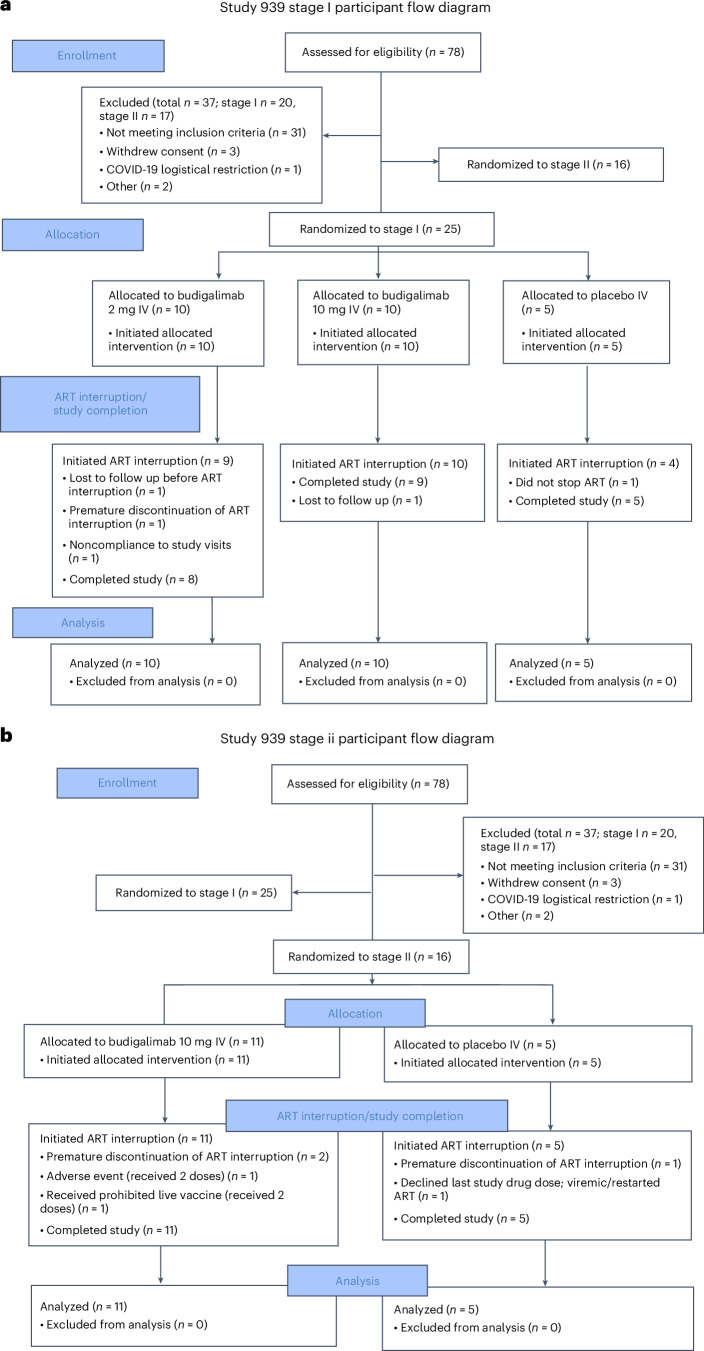
Full CONSORT participant flow diagram for Study M19-939 **a**) stage I and **b**) stage II. ART, antiretroviral therapy; IV, intravenous.

An abridged participant disposition is outlined in Table 1, with a full CONSORT flow diagram in Fig. 1 Eligible participants were adult PLWH receiving suppressive ART (plasma HIV-1 RNA < 20 copies ml^−1^ for the past ≥6 months) with a CD4^+^ T cell count ≥500 cells µl^−1^, a CD4^+^ T cell nadir of ≥200 cells μl^−1^ during chronic infection, no evidence of early ART initiation (<3 months) after acute infection and no history of AIDS. In both stages, 78 PLWH were screened, and a total of 41 participants were enrolled and received ≥1 dose of study drug. Participants were enrolled from 11 sites in the United States (9 sites), Canada (1 site) and Australia (1 site). The first participant visit occurred 30 January 2020, and the last participant visit occurred 27 February 2023. Participant demographics and baseline characteristics were balanced across treatment groups with representation of eligibility criteria, except for sex, which was predominantly male. All participants identified as cisgender (Table 2).

**Table 1:** Participant disposition

	Stage I			Stage II	
	Placebo (*n* = 5) *n*/*N*	2 mg IV (*n* = 10) *n*/*N*	10 mg IV (*n* = 10) *n*/*N*	Placebo (*n* = 5) *n*/*N*	10 mg IV (*n* = 11) *n*/*N*
Randomized	5/5	10/10	10/10	5/5	11/11
Treated	5/5	10/10	10/10	5/5	11/11
Initiated ATI	4/5^a^	9/10^b^	10/10	5/5	11/11
Restarted ART^c,d^	4/4	8/9	10/10	5/5	9/11
Premature discontinuation of study treatment	0	1/10^b^	0	1/5^e^	2/11 ^f^
Completed study treatment	5/5	9/10	10/10	4/5	9/11
Premature discontinuation of study	0	2/10^bg^	1/10 ^g^	0	0
Completed the study	5/5	8/10	9/10	5/5	11/11

^a^Participant did not stop ART.

^b^Participant discontinued study owing to noncompliance with study procedures after receiving 1 study drug dose.

^c^Denominator is participants who initiated ATI.

^d^ART-restart criteria were ≥1 of the following: ≥2 consecutive plasma HIV-1 RNA measures ≥1,000 copies ml^−1^ for ≥4 weeks; ≥2 consecutive measures of >30% decline from baseline CD4^+^ T cell count or absolute CD4^+^ T cell count <350 cells µl^−1^; HIV-associated symptoms, including symptoms of retroviral rebound syndrome; pregnancy; or participant or investigator request to reinitiate ART.

^e^Participant chose to not receive last infusion owing to viremia and lack of benefit from treatment.

^f^One participant received prohibited live vaccination after two doses of study drug; one participant experienced an AE (grade 1 reversible hyperthyroidism) after two doses of study drug.

^g^Participant lost to follow-up after receiving all doses of study drug.

ART, antiretroviral therapy; ATI, analytical treatment interruption, IV, intravenous.

**Table 2:** Baseline characteristics

	Stage I			Stage II	
Characteristic	Placebo (*n* = 5)	2 mg IV (*n* = 10)	10 mg IV (*n* = 10)	Placebo (*n* = 5)	10 mg IV (*n* = 11)
Sex – male^a^, *n*/*N*	5/5	10/10	10/10	5/5	10/11
Race, *n*/*N*					
White	5/5	8/10	7/10	5/5	8/11
Black/African American	0	2/10	3/10	0	3/11
Ethnicity, *n*/*N*					
Hispanic/Latino	3/5	5/10	3/10	2/5	3/11
Not Hispanic/Latino	2/5	5/10	7/10	3/5	8/11
Age, median (min, max), y	44 (24, 57)	38.5 (26, 60)	49.5 (22, 65)	46 (38,63)	48 (29, 64)
BMI, median (min, max), kg/m^2^	24.7 (20.3, 29.0)	24.9 (22.5, 30.9)	26.1 (23.2, 31.1)	28.3 (22.5, 32.7)	27.9 (21.7, 34.5)
Duration of HIV-1 diagnosis, median (min, max), y	6 (1, 32)	5 (1, 26)	12.5 (1, 28)	16 (11, 26)	10 (3, 24)
CD4^+^ T cell count, median (min, max), cells $\mu$ l^−1^	651 (513, 880)	759.5 (655, 1416)	783.5 (470, 990)	733 (449, 838)	759 (428, 1041)
CD4^+^ T cell nadir, median (min, max), cells $\mu$ l^−1^	350 (253, 380)	390 (212, 781)	374 (350, 1037)	401 (180, 500)	372 (88, 529)
Pre-ART viral load setpoint, median (min, max), copies ml^−1^	53,269 (3148, 136,892)^b^	34,970.5 (9588, 98,257)^c^	406,250 (14510, 10,000,000)^d^	361,000 (38,000, 684,000)^e^	33,810 (20, 544,000)^f^
Duration of viral suppression on ART, median (min, max), y	6.1 (1.5, 31)	5.1 (1.8, 15)	12.0 (1.7, 28)	15.0 (11, 23.3)	10.0 (3.3, 24.3)
ART regimen at study entry, *n*/*N*					
PI based	0	1/10	1/10	0	0
INSTI based	5/5	9/10	9/10	5/5	10/11
Other	0	0	0	0	1/11^g^
History of resistance on ART – yes, n/N	1/5^h^	0	0	0	0^i^

^a^All participants identified as cisgender

^b^*n* = 3

^c^*n* = 8

^d^*n* = 4

^e^*n* = 2

^f^*n* = 5

^g^NNRTI-based regimen;

^h^*n* = 1 resistance to NNRTI

^i^*n* = 10.

ART, antiretroviral therapy; BMI, body mass index; INSTI, integrase strand transfer inhibitor; IV, intravenous; NNRTI, non-nucleoside reverse transcriptase inhibitor; PI, protease inhibitor.

The primary endpoints were safety and tolerability including drug-related grade ≥3 AEs, study drug-related AEs, IRAEs and AEs corresponding to retroviral rebound syndrome and the pharmacokinetics of repeat dosing of budigalimab in PLWH while on suppressive ART and during ATI. There were no secondary endpoints, and exploratory analyses evaluated peripheral PD-1 receptor saturation, the impact of the regimen on immune responses (induction of proliferation and activation markers, serum cytokine/chemokines levels, ex vivo HIV-specific T cell response), impact of the regimen on latency reversal and the size of the HIV reservoir, and the impact on viral load kinetics. Impact of the regimen on serum cytokine/chemokine levels and latency reversal were pre-planned exploratory analyses and will be presented elsewhere. Additionally, the size of the HIV reservoir was originally planned as an exploratory analysis, but a limitation of this study was lack of sample availability so therefore this will not be presented.

### Primary endpoint: Safety

AEs were monitored throughout the study to a total of 44 weeks in stage I and 36 weeks in stage II. A total of 29/41 (70.7%) participants experienced ≥1 AE(s), primarily (*n* = 27) grade 1 to 2 in severity (Table 3). There were no treatment-related AEs above grade 3, and three grade 3 AEs were experienced between two participants. In both participants, the events occurred in stage I (*n* = 1, 2 mg group; *n* = 1, placebo group) and were deemed unrelated to treatment. Two participants receiving budigalimab 10 mg in stage I or II experienced one reversible grade 1 IRAE, deemed in each case as related to the study drug. In stage I, one participant developed thyroiditis, which was observed on study day 59 and resolved on day 142. The participant was monitored and completed the study. In stage II, one participant experienced hyperthyroidism during treatment (observed on study day 3 and resolved on day 71), and the study drug was withdrawn. One participant receiving budigalimab 10 mg in stage II experienced mild retroviral rebound syndrome. There were no treatment-related hepatic AEs of special interest (AESIs), no infusion-related reaction AEs and no deaths in the study. With 10 mg Q2W×4 dosing in stage II, common treatment-related AEs occurring in ≥2 participants were fatigue (*n* = 3) and diarrhea, malaise and nausea (*n* = 2 each). Across all treatment arms, median CD4^+^ T cell counts showed modest declines from baseline at week 28 (Extended Data Fig. 1), but no trends were apparent in CD4^+^ and CD8^+^ T cell counts from baseline to post-baseline in a shift analysis of stages I and II based on the Division of AIDS Table for Grading the Severity of Adult and Pediatric Adverse Events (DAIDS) criteria, and/or categorization using laboratory-specific ranges (low, normal, or high) and/or CD4^+^ T cell-related AEs. There was no occurrence of AIDS-defining illness during the study.

**Table 3:** Safety assessment

	Stage I			Stage II	
	Placebo (*n* = 5) *n*/*N*	2 mg IV (*n* = 10) *n*/*N*	10 mg IV (*n* = 10) *n*/*N*	Placebo (*n* = 5) *n*/*N*	10 mg IV (*n* = 11) *n*/*N*
Any AE	2/5	6/10	6/10	4/5	11/11^a^
TRAE^b^	0	2/10	2/10	3/5	5/11
Serious AE	1/5^c^	0	0	0	0
Serious TRAE	0	0	0	0	0
TRAE leading to discontinuation	0	0	0	0	1/11
AE grade ≥3	1/5 ^d^	1/10^e^	0	0	0
TRAE grade ≥3	0	0	0	0	0
IRAE	0	0	1/10^f^	0	1/11^g^
Death	0	0	0	0	0

^a^One report of retroviral rebound syndrome.

^b^As assessed by investigator.

^c^Rectal hemorrhage after rectal biopsy.

^d^Rectal bleeding.

^e^Increased amylase; increased lipase.

^f^Reversible grade 1 thyroiditis (days 59–142).

^g^Reversible grade 1 hyperthyroidism (days 3–71).

AE, adverse event; IRAE, immune-related adverse event; TRAE, treatment-related adverse event.

### Primary endpoint: Pharmacokinetics

**Fig. 2:**
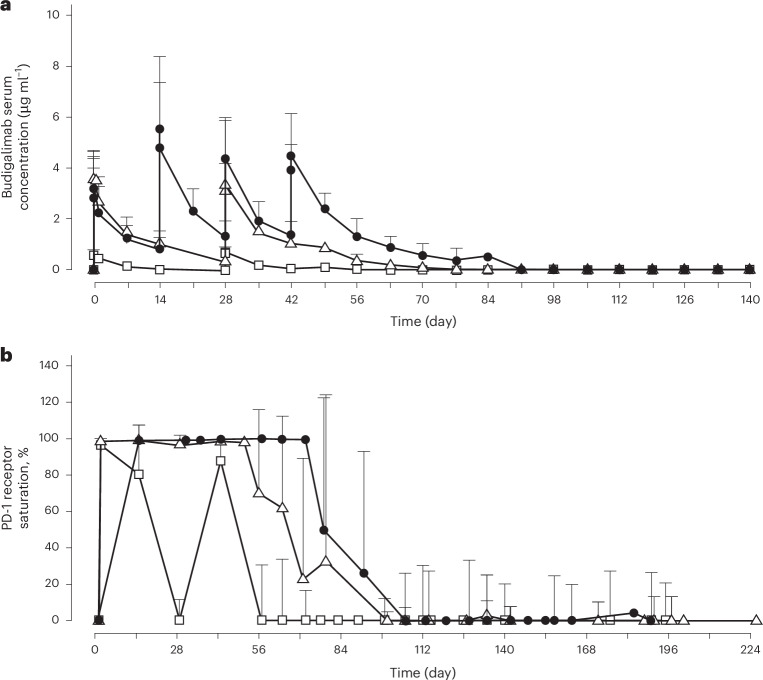
Budigalimab pharmacokinetics and pharmacodynamics. **a**,**b**, Serum concentration–time profiles of budigalimab (**a**) and PD-1 receptor saturation on CD8^+^ T cells (**b**), as measured by percent PD-1 receptor saturation, with repeated IV dosing. Squares represent mean (+standard deviation (s.d.)) values with two doses of 2 mg IV Q4W (stage I; *n* = 10). Triangles represent mean (+s.d.) values with two doses of 10 mg IV Q4W (stage I; *n* =10). Black circles represent mean (+s.d.) values with four doses of 10 mg IV Q2W (stage II; *n* = 11). IV, intravenous; PD-1, programmed cell death 1; Q2W, every 2 weeks; Q4W, every 4 weeks.

Budigalimab concentration–time profiles were assessed for the Q4W×2 (stage I) and Q2W×4 (stage II) regimens (Fig. 2a). In stage I, the geometric mean maximum observed serum concentrations (*C*_max_) were 0.602 and 0.684 μg ml^−1^ after the first and second doses of budigalimab 2 mg and 3.57 and 3.2 μg ml^−1^ after the first and second doses of budigalimab 10 mg (Extended Data Table 2). Maximum budigalimab serum concentrations were observed at ~1–3 h after dose initiation. The terminal elimination half-life was ~3–5 days with 2 mg dosing and 6 days with 10 mg dosing. In stage I, consistency of budigalimab exposures between dose 1 (with participants on ART) and dose 2 (with participants starting ATI) suggested no apparent impact of ART or viremia over a dosing interval of 4 weeks. Using an analysis of covariance, dose-normalized values for the area under the serum concentration–time curve (AUC_tau_) were observed to be statistically different between budigalimab 2 mg and 10 mg during each dosing interval (Extended Data Table 3), with the results suggesting that exposures following 10 mg were more than dose proportional as compared with 2 mg. Analysis of repeated measures of *C*_max_ and AUC_tau_ indicated negligible accumulation of budigalimab with Q4W dosing in stage I.

Administration of four doses of budigalimab 10 mg Q2W was selected for stage II and resulted in a *C*_max_ of 3.10 μg ml^−1^ after the first dose and 4.35 μg ml^−1^ after the fourth dose (Extended Data Table 4). Maximum budigalimab serum concentration was observed at a median of ~1–2 h from infusion start, and the terminal elimination half-life was ~8 days. A 1.5-fold to 2-fold drug accumulation, based on *C*_max_ and AUC_tau_ respectively, was observed following the last dose compared with the first dose after four doses of budigalimab 10 mg Q2W.

Finally, treatment-emergent antidrug antibodies (ADAs) were observed in ~52% of participants in the active treatment arms; 4/10, 5/10 and 7/11 of participants receiving budigalimab 2 mg (stage I), 10 mg (stage I) and 10 mg (stage II), respectively, with a baseline incidence of 0% for all groups. Across all treatment arms in stages I and II, the exposures of participants with positive ADA titers largely overlapped with those of participants with negative ADA titers, suggesting no apparent impact of ADA status on budigalimab pharmacokinetics.

### Exploratory: PD-1 receptor saturation

Expression of PD-1 receptors on CD4^+^ and CD8^+^ T cells and subsets in blood at baseline were comparable across dose groups. PD-1 receptor saturation was examined in tandem with budigalimab serum concentration–time profiles, with 95% receptor saturation considered nearly complete saturation owing to the parameters of the assay. With budigalimab 2 mg dosing, >95% PD-1 receptor saturation on CD8^+^ T cells, was observed for <15 days (Fig. 2b). With 10 mg dosing, >95% receptor saturation was observed for a median of 29 days (stage I) and 30 days (stage II). The differences in PD-1 receptor saturation between budigalimab 2 mg and 10 mg are consistent with the findings that budigalimab serum concentrations were sustained throughout the 4-week dosing interval with 10 mg vs 2 mg in stage I, with more than dose-proportional exposures observed with budigalimab 10 mg relative to 2 mg.

The total period of ≥95% PD-1 receptor saturation was ~70 days with budigalimab 10 mg Q2W×4 doses. Serum concentration of ~0.5 µg ml^−1^ was associated with near complete (≥95%) PD-1 receptor saturation based on the pharmacokinetic and pharmacodynamic profiles of budigalimab.

### Exploratory: ATI, viral load kinetics and ART restart

**Fig. 3:**
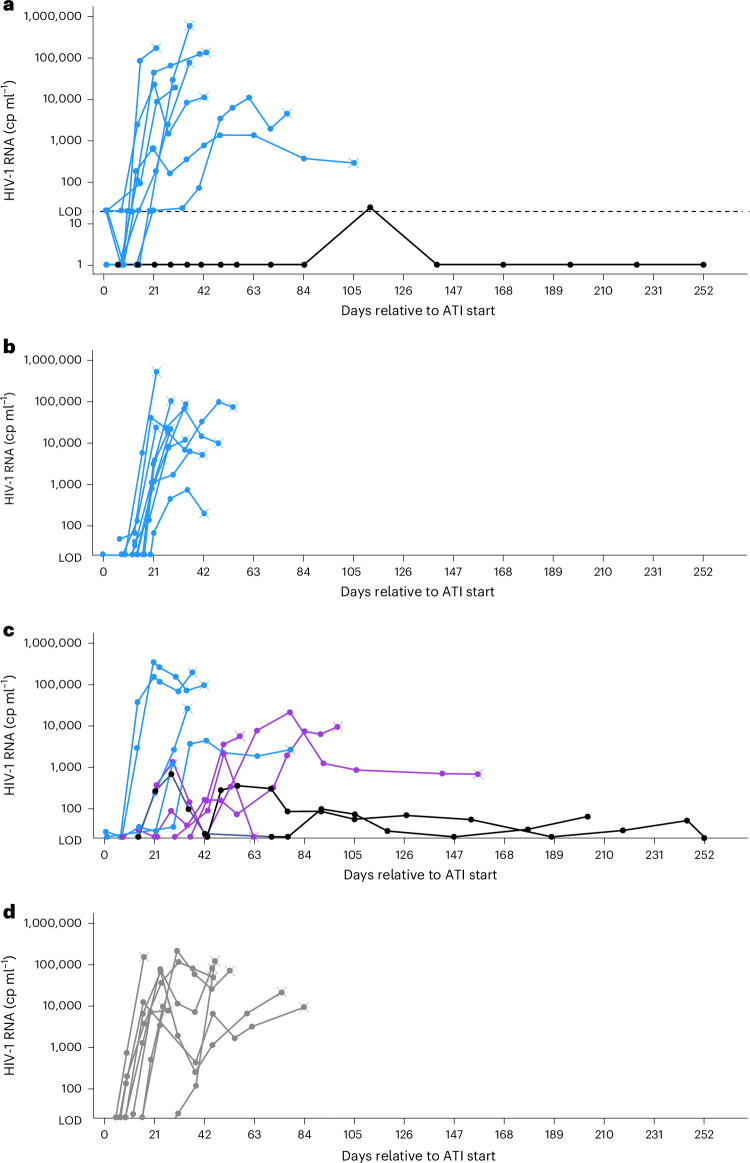
Viral load kinetics during ATI. **a**–**d** Individual plasma HIV-1 RNA levels (log scale) of participants receiving (**a**) two doses of budigalimab 2 mg IV Q4W with ATI starting at week 4 with the second dose (*n* = 9; one participant maintained a viral load <50 copies ml^−1^ through ATI to study end); (**b**) two doses of budigalimab 10 mg IV Q4W with ATI starting at week 4 with the second dose (*n* = 10); (**c**) four doses of budigalimab 10 mg IV Q2W with ATI starting on day 0 with the first dose (*n* = 11; two participants discontinued study drug: one for protocol violation (prohibited live vaccination) and one for AE (grade 1 reversible hyperthyroidism)); and (**d**) placebo in stage I or stage II (*n* = 9). Blue lines represent participants taking budigalimab and not exhibiting a delay in viral rebound (>21 days) and/or off-ART viral control (<200 copies ml^−1^); purple and black lines represent participants taking budigalimab and experiencing a delay in viral rebound and/or off-ART viral control, with black lines differentiating the participants who remained off ART for entire study period; and gray lines represent participants receiving placebo. Data points marked with an X represent the last observed point before reinitiation of ART. Dashed line represents LOD of viral load assay (20 copies ml^−1^). ART, antiretroviral therapy; ATI, analytical treatment interruption; cp, copies; IV, intravenous; LOD, limit of detection; Q2W, every 2 weeks; Q4W, every 4 weeks.

Participants initiated ATI with close monitoring of HIV-1 RNA viral load. In stage I, ATI commenced at week 4 (ie, immediately after the second dose). In stage II, ATI commenced on day 1 with the first dose. ATI was planned for 12 weeks unless ART-restart criteria (Methods) were met. Median (interquartile range) time to HIV-1 RNA ≥ 200 (defined as viral rebound) and ≥1,000 copies ml^−1^ (ART-restart threshold) was 21 (21–24) days and 21 (21–28) days, respectively, for the combined stage I/stage II placebo group (*n* = 10). In the budigalimab 10 mg Q2W×4 group (*n* = 11), the median (interquartile range) time to HIV-1 RNA viral load ≥200 and ≥1,000 copies ml^−1^ was 29 (21–50) days and 36 (29–77) days, respectively. All but one participant experienced viral rebound (HIV-1 RNA to ≥200 copies ml^−1^) during ATI in both stages (Fig. 3a–d): a participant receiving budigalimab 2 mg Q4W×2 maintained HIV-1 RNA < 50 copies ml^−1^ off ART through study end (baseline characteristics, Extended Data Table 5).

**Extended Data Fig. 2:**
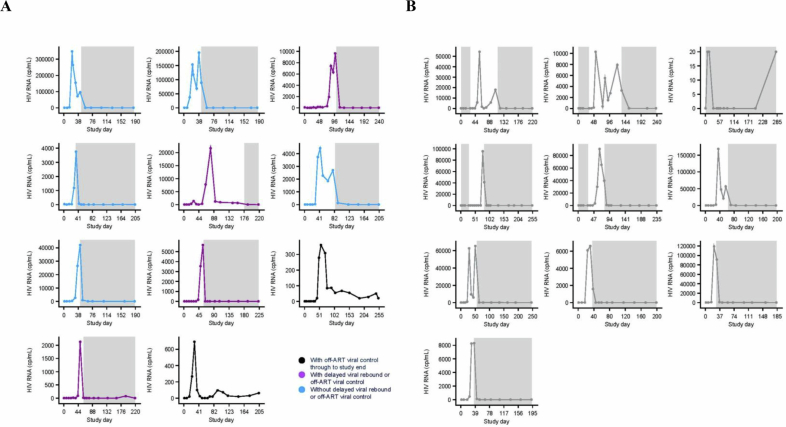
HIV-1 RNA curves over entire study for each participant receiving **A**) budigalimab 10 mg IV Q2W×4, and **B**) placebo (combined stage I and stage II). Note variation in y-axis scale. Gray boxes represent RNA values collected outside of analytical treatment interruption. ART, antiretroviral therapy; cp, copies; IV, intravenous; Q2W, every 2 weeks.

In an exploratory analysis of viral load kinetics in stage II, the group (*n* = 11) receiving budigalimab 10 mg Q2W×4 experienced a trajectory and magnitude of viral load kinetics that varied considerably among participants (Fig. 3c and Extended Data Fig. 2). For 5 of 11 participants, viral load rebounded and remained elevated (leading to the reinitiation of ART), and of these, two had not received the full schedule of budigalimab doses. However, a group of six participants (baseline characteristics, Extended Data Table 5) experienced a low viral load peak, with delayed viral rebound (>21 days) and/or off-ART viral control (<200 copies ml^−1^) for ≥6 weeks after ATI. Budigalimab exposures of participants with or without viral load control were comparable. Among the 6 participants, 2 met ART-restart criteria and reinitiated ART during the 12 weeks of planned ATI. Four participants did not meet ART-restart criteria during ATI based on viral load, CD4 count or clinical symptoms, with two of the four reinitiating ART on request. The other two participants sustained ART-free viral control (HIV-1 RNA < 200 copies ml^−1^) until study end (204 and 252 days). No participants in the pooled placebo group demonstrated viral control after rebound during ATI (Fig. 3d), and median peak HIV-1 RNA was numerically higher with placebo (65,400 copies ml^−1^) than with budigalimab 10 mg Q2W×4 (9,640 copies ml^−1^). In stage II, median (range) time to viral resuppression following ART restart for budigalimab 10 mg and placebo was 46 (10–110) days and 52 (44–107) days, respectively. No participant in stage II experienced virologic failure, and no change from baseline ART regimen was needed owing to the emergence of drug-resistant virus.

Overall, reasons for ART restart in the nine stage II participants receiving budigalimab who restarted ART were participant or investigator request (*n* = 6); meeting HIV-1 RNA viral load criterion (*n* = 3); meeting CD4^+^ T cell count criterion (*n* = 2); or symptoms associated with HIV, including retroviral rebound syndrome (*n* = 1). Testing for individual antiretroviral drugs (abacavir, bictegravir, cobicistat, dolutegravir, emtricitabine, lamivudine, tenofovir) identified two participants in the 10 mg stage II group receiving budigalimab with one time point of quantifiable antiretroviral drug in serum followed by continuous absence of antiretroviral drug while off ART; these were considered artifactual blips. For the two participants remaining off ART until the end of the study, medical history confirmed that they were not previous elite controllers.

### Exploratory analysis: T cell phenotypic assessment

**Extended Data Fig. 3:**
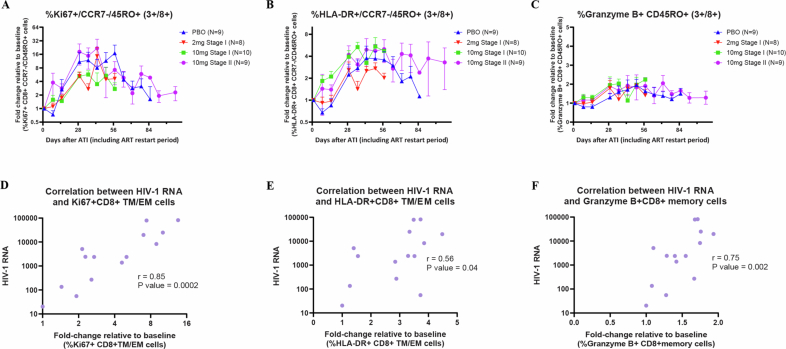
Expression of T cell activation and proliferation markers. Change in the expression of proliferation and activation markers **A**) Ki67, **B**) HLA-DR, and **C**) Granzyme B+ on CD8+ memory cells relative to baseline and correlation between plasma HIV RNA and **D**) Ki67, **E**) HLA-DR, and **F**) Granzyme B in stage I and stage II of the study. Spearman correlations were conducted grouping the entire dataset. T cell memory subsets were assessed using markers CD45RO and CCR7. Naive cells were identified as CD45RO − CCR7 + , central memory cells were identified as CD45RO + CCR7 + , TM/EM cells were identified as CD45RO + CCR7 − , TE cells were identified as CD45RO − CCR7− cells, and memory cells were identified as CD45RO+ cells. HLA-DR, human leukocyte antigen DR; Ki67, markers antigen Kiel 67; PBO, placebo; TM/EM, transient/effector memory.

**Extended Data Fig. 4:**
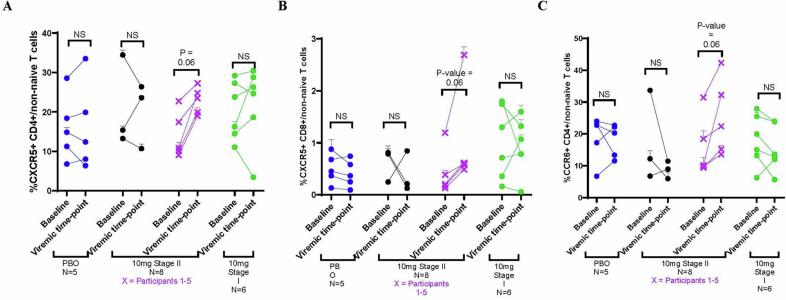
T cell subsets during ATI. Immunophenotyping at baseline and during a viremic time point indicating changes in **A**) T follicular helper-like (TFH) cells, **B**) peripheral CXCR5 + CD8 + T cells, and **C**) CCR6 + CD4 + T cells. Crosses indicate the 5 of the 6 participants in the stage II budigalimab 10-mg Q2W×4 arm who experienced a delayed viral rebound with a low viral load and/or viral load resuppression after rebound. P-values were assessed using Wilcoxon matched-pairs signed rank test. Viral load in the post-baseline samples ranged between undetectable and 81,950 copies/mL in the 10-mg Q4W×2 arm, between 405 and 54,400 copies/mL in the PBO arm, and between 22 and 96,400 copies/mL in the 10-mg Q2W×4 arm. Nearly complete PD-1 receptor saturation with budigalimab ( > 95 %) was observed on CD4+ and CD8 + T cells at the post-baseline sample in both 10-mg treatment arms (data not shown). Non-naive CD4+ and CD8 + T cell populations were identified using markers CD45RO, CD28, and CCR7, wherein naive cells were identified as CD28 + CD45RO − CCR7+ cells and a Boolean filter was utilized to distinguish non-naive cells. Although, Th1 is defined as CCR4 − CCR6 − CXCR3+ cells, Th2 as CCR4 + CCR6 − CXCR3− cells, Th17 as CCR6 + CD161+ or CCR6+ cells, and TFH-like as CXCR5+ cells, CXCR3+ cells could not be gated appropriately. Hence, within CD4 + T cell non-naive cells, cells were gated with CCR4 and CCR6 markers. At baseline, 71% ± 14% of the CD8 + /non-naive/CXCR5+ cells expressed PD-1 and 42% ± 8% of TFH-like cells expressed PD-1 (data not shown). No changes in CD4 + /non-naive/CCR4+ cells were apparent at post-baseline time points (data not shown). ATI; analytical treatment interruption; PBO, placebo; Q4W, every 4 weeks.

In stage II, T cell activation and proliferation, and changes in immune cell subpopulations were evaluated during the ATI period. The percentage of CD8^+^ T cells expressing activation and proliferation markers Ki67, human leukocyte antigen DR (HLA-DR) and Granzyme B increased on memory cells (CD45RO^+^), especially transient/effector memory subset cells in all treatment groups and PBO (Extended Data Fig. 3a–c). Increases in T cell Ki67 proliferation marker and activation markers HLA-DR and Granzyme B across the study correlated with HIV RNA (Extended Data Fig. 3d–f). No budigalimab-related changes in T cell activation or proliferation were apparent. In a limited flow cytometric analysis of T cell subsets, trends were observed in budigalimab-mediated expansion of peripheral CXCR5^+^ CD8^+^ T cells (*P* = 0.06), TFH-like cells (*P* = 0.06), and CCR6^+^ CD4^+^ T cells (Th17; *P* = 0.06) in participants receiving budigalimab 10 mg Q4W×2 who had delayed viral rebound and/or off-ART viral control (Extended Data Fig. 4).

In a limited analysis, an increase in HIV-specific T cell response to HIV gag peptide pools was observed in a subset of samples, and these changes were correlated with viral load and associated increases in CD8^+^ T cell counts and T cell activation/proliferation (Supplementary Fig. 1). No specific association between HIV-specific functional responses and budigalimab treatment or efficacy response were apparent. A limitation of this assay is the utilization of HIV peptide pools to measure T cell responses does not account for immune escape variants and is therefore not a true estimate of autologous T cell responses; additionally, ATI-associated T cell activation and changes in CD4^+^ and CD8^+^ T cell counts during viral rebound may confound assay interpretation.

### Post hoc analysis: HLA genotyping

HLA typing was available for 36 of 41 participants in the study (Extended Data Table 6). Twelve participants had protective alleles, HLA-B*13:02, HLA-B*14:02, HLA-B*27:05, HLA-B*40:02, HLA-B*52:01, HLA-B*57:01, HLA-B*57:03 or HLA-B*58:01. These HLA class I alleles are proposed to select for HIV epitopes that are derived from highly constrained and difficult-to-mutate sites within the HIV proteome^37–39^. Eighteen participants had HLA alleles (HLA-B*07:02, HLA- B*8:01, HLA-B*18:01, HLA-B*35:01, HLA-B*35:02, HLA-B*38:01, HLA-B*40:01 or HLA-B*55:01) that are associated with disease progression^37,38^. Four participants had alleles associated with protection as well as progression. No specific associations between HLA alleles and viral load were apparent in this small dataset.

## Discussion

In this phase 1b study in PLWH, low-dose budigalimab administered for a finite duration was well tolerated, and pharmacokinetic assessment supported 10 mg IV Q2W dosing. Safety and pharmacokinetics of a single dose of budigalimab by IV or subcutaneous injection have also been evaluated in PLWH (NCT04799353), and together, the results support further study of the drug. A phase 2 efficacy, safety and pharmacokinetic study with ATI of budigalimab alone or combined with the anti-α4β7 mAb ABBV-382 in PLWH is underway (NCT06032546).

Previous phase 1 studies of budigalimab in patients with advanced solid tumors administered substantially higher doses, 250 mg Q2W to 500 mg Q4W until disease progression^34^, than doses proposed in the current study in PLWH. With the higher doses in oncology setting, all participants experienced ≥1 AE, with >60% reporting grade ≥3 events and ~60% reporting AEs deemed related to treatment, most commonly hypothyroidism, diarrhea, fatigue and pruritis^34^. This profile is similar to other anti-PD-1 therapies for oncology indications. The current study reported a more favorable safety profile that might be expected with substantially lower doses (≤10 mg) over a finite period and a subsequently restricted period of PD-1 receptor saturation, in a different population, namely PLWH. Shorter immune checkpoint dosing duration has previously been reported to be associated with reduced grade 3 and 4 IRAEs^40^. Overall, 71% of participants experienced ≥1 AE, with >90% of those being grade 1 or 2. Two participants experienced grade 3 AEs, which were deemed unrelated to budigalimab. There was one serious AE, also deemed unrelated to treatment, and no deaths occurred during the study. With respect to immune-related events, a single reversible and mild IRAE was reported in each of two participants receiving budigalimab treatment: one case of hyperthyroidism, which led to drug discontinuation, and one case of thyroiditis. Although thyroid dysfunction is a known IRAE with PD-1-blocking therapies in oncology and has been studied for risk factors, pathogenesis, clinical course and treatment outcomes^41–44^, limited data on these two events precluded further analysis. There were no budigalimab-related hepatic AEs, no infusion-related reaction AEs and no budigalimab-related injection reaction AEs in the current study. The closely monitored ATI was also well tolerated by participants, including in participants receiving placebo. There was one mild AE of retroviral rebound syndrome that resolved with ART restart.

With budigalimab 10 mg IV Q2W, the terminal elimination half-life was ~8 days. Budigalimab exposures were more than dose proportional at 10 mg IV compared with 2 mg IV, and up to a twofold serum drug accumulation was observed with 10 mg Q2W after four doses. With the budigalimab 10 mg IV Q2W (four doses) regimen, serum drug concentrations maintained near complete (>95%) PD-1 receptor saturation on CD8 + T cells for ~10 weeks post ATI initiation. In contrast, 2 mg dosing was not effective at achieving sustained elevated budigalimab serum concentrations or receptor saturation, and the dose proportionality assessment suggested nonlinear pharmacokinetic characteristics of target-mediated drug disposition with dosing of 2 mg IV.

Delayed viral rebound and/or evidence of off-ART viral control was observed in six of nine participants completing treatment with four 10 mg biweekly doses, with two participants remaining off ART to study end (204 and 252 days). Within the context of other published results, the result of 2 of 9 (22.2%) participants with extended viral control observed here is numerically higher than results seen in other studies involving nonprimary infection post-treatment controllers, which have reported off-ART control (<50, <400 or <1,000 copies ml^−1^) among 2.5% to 7.4% of participants (*N* = 33 to >600) for >24 weeks^45–49^. Even in a highly selected population of ten PLWH with chronic HIV, excellent immune status, very low HIV reservoir and durable viral suppression, only one (10%) exhibited off-ART viral control (<400 copies ml^−1^) up to week 48 (ref. ^50^). The phase 1 viral kinetic results presented here are exploratory and cannot conclude the off-ART control is caused by the administration of budigalimab. Nevertheless, multiple assessments have not identified a plausible alternative explanation for the kinetics observed with 10 mg biweekly doses. In addition, none of the participants in the pooled placebo group demonstrated viral control after rebound during ATI, and median peak HIV-1 RNA was numerically higher for placebo compared with budigalimab 10 mg Q2W×4. This expected rapid viral rebound in the placebo group and a lack of any impact with the budigalimab 2 mg dosing, which resulted in incomplete PD-1 RS, suggest that the delayed viral load kinetics observed with 10 mg dosing may be an outcome of drug action.

Baseline characteristics including age, duration of HIV infection, duration of viral suppression on ART, pre-ATI ART regimen, CD4^+^ and CD8^+^ T cell counts, and PD-1 expression in the seven participants with altered viral load kinetics were representative of the study population. Viral rebound during ATI was associated with T cell activation and proliferation, with no specific association with budigalimab treatment being apparent. In an exploratory analysis of T cell subset distribution, trends in budigalimab-mediated expansion of peripheral CXCR5 + T cells and CCR6^+^ CD4^+^ T cells (Th17-like cells) were observed in participants who had delayed viral rebound and/or off-ART viral control in budigalimab 10 mg Q2W×4 group. PD-1 blockade has been associated with increased expression of CXCR5^+^ Granzyme B^+^ CD8^+^ T cells in SIV studies in primates in both peripheral blood and lymphoid tissues, signifying expansion of T cells with effector-like properties with both proliferative and cytotoxic potential^30,31^. CXCR5 expression on CD8^+^ T cells could potentially enable migration of these cells into the HIV reservoir–rich germinal center of B-cell follicles and be an important contributor to control of viral replication. A trend in budigalimab-mediated expansion of peripheral CCR6^+^ CD4^+^ Th17-like cells was also observed in the participants with low viral load in the budigalimab 10 mg Q2W×4 group. PD-1 blockade in the SIV model has been associated with reconstitution of Th17 cells in rectal mucosal tissue, potentially contributing to mucosal immunity^30^.

The current biomarker analyses are limited by the small dataset and lack of available samples for further longitudinal analyses. A lack of available samples also precluded evaluation of the size of the HIV reservoir and potential correlates with viral load kinetics. Other biomarkers collected during the study will be published in a separate report. The study population size was small, as is typical for a phase 1 study. In addition, almost all study participants were male and all participants identified as cisgender, which does not reflect the diversity of PLWH. Although the study was open to people of all sexes and genders, the need for contraception and the burden from the number of visits required for intensive monitoring during the ATI period may have contributed to lower female enrollment. The lack of enrollment of non-cisgender may reflect the general people living with HIV in care in the study sites. However, given the early-phase nature of this small safety and pharmacokinetic study, the historical tendency toward inclusion of male participants may limit variability due to hormonal fluctuations associated with the menstrual cycle. Relatedly, assessments of viral load kinetics were exploratory, and this phase 1 study was not powered to undertake an efficacy analysis. Also, as may be expected in a study with ATI, there were relatively high rates of ART restart by participant or investigator request, 12 participants (54.5%) and 9 participants (64.3%) in stages I and II, respectively. Although voluntary ART restart is essential to mitigate safety risks, including the risk of HIV transmission, these requested restarts limit interpretation of some measures, such as the median time to rebound. A phase 2 study currently underway has considered these findings in the determination of an appropriate sample size as well as implementation measurements, including site selection criteria to increase inclusion of females and non-cisgender PLWH.

In conclusion, this study evaluated budigalimab doses that are substantially lower than those dosed for oncologic indications with the goal of improving tolerability and safety among PLWH. A finite dosing regimen with four IV doses of budigalimab 10 mg Q2W was associated with favorable safety and pharmacokinetic profiles and with no new safety signals detected in PLWH, supporting further evaluation. The frequency of IRAEs and severe IRAEs appears to be reduced with these lower doses and finite duration and PD-1 receptor saturation regimen when administered in a population of asymptomatic PLWH. An exploratory analysis of efficacy showed the potential for delayed viral rebound and/or off-ART viral control. Together, these findings indicate further study of budigalimab in this population is warranted, potentially in combination with other immunotherapies, and may contribute to advances in effective strategies for durable off-ART control and, potentially, HIV cure.

## Methods

### Ethical approval

Sites used a central institutional review board (Advarra) or local ethics committees to have the study protocol, informed consent, and participant information approved. The study was conducted in accordance with International Council for Harmonisation (ICH) guidelines, and applicable regulations, guidelines, and principles had their origin in the Declaration of Helsinki. Written informed consent, including a full discussion of risks of budigalimab, risks of ATI and conditions for ART reinitiation, was obtained for each participant before screening or study-specific procedures. There was no external safety board as this was a phase 1 study and the sponsor was unblinded to the data. Investigators and sites were also unblinded to the HIV RNA and CD4 monitoring so they could make prompt decisions on ART restart. Participants were compensated according to local laws and fair market compensation for a phase 1 study.

### Study design and treatments

Study M19-939 was a phase 1b, randomized, double-blind, multicenter placebo-controlled study to evaluate the safety, pharmacokinetics and pharmacodynamics of multiple doses of budigalimab in PLWH with ART-suppressed viral load, including a planned ATI (CTN.gov registration: NCT04223804). A conservative, gated two-stage approach evaluated two low doses of budigalimab with satisfactory stage I results of safety, pharmacokinetics and pharmacodynamics required before initiating evaluation of a longer dosing regimen in stage II. Sample sizes and dosing strategies achieved adequate safety, pharmacokinetic and pharmacodynamic profiling while offering flexibility to limit overall drug exposure and potential negative effects, as needed. In stage I, participants were randomized (1:2:2) to placebo, budigalimab 2 mg, and budigalimab 10 mg administered IV Q4W×2, with doses at day 1 and week 4. Following the second dose on week 4, participants started a closely monitored ATI planned for 12 weeks, with final duration based on ART-restart criteria. ART was to be restarted after the ATI, with follow-up to a total study duration of 44 weeks. Preliminary data from stage I were used to inform dosing in stage II. In stage II, participants were randomized in parallel (1:2) to placebo or budigalimab 10 mg IV for Q2W×4 at day 1, and weeks 2, 4 and 6. At day 1, after the first dose, participants started a closely monitored ATI planned for 12 weeks, with final duration based on ART-restart criteria. ART was to be restarted after the ATI, with follow-up to a total study duration of up to 36 weeks.

The inclusion of an ATI aligned with expert panel recommendations^51^ and multiple mitigation strategies were employed to reduce risk to participants and their partners. The ATI was used to evaluate budigalimab safety in the setting of viral rebound and in the absence of ART, which will be necessary for studying ART-free viral remission of future treatments. Moreover, dosing with budigalimab may protect against negative immune effects induced by rebound viremia, and viral rebound during ATI may be required to elicit robust T cell responses following dosing and to evaluate HIV-specific pharmacodynamic effects. Viral rebound during ATI was defined as a single plasma HIV-1 RNA ≥200 copies ml^−1^; however, this viral load threshold was not an ART-restart criterion. The specific criteria for reinitiation of ART during ATI were ≥1 of the following: ≥2 consecutive plasma HIV-1 RNA measures ≥1,000 copies ml^−1^ for ≥4 weeks; ≥2 consecutive measures of >30% decline from baseline CD4^+^ T cell count or absolute CD4^+^ T cell count <350 cells µl^−1^; HIV-associated symptoms, including symptoms of retroviral rebound syndrome; pregnancy; or participant or investigator request to reinitiate ART.

### Participants, randomization, and blinding

Eligible participants were female or male sex (of any gender; sex and/or gender were self-reported), 18 to 65 years old with a positive anti-HIV-1 antibody test and negative anti-HIV-2 antibody test results on ART for ≥12 months (current regimen ≥8 weeks) before screening and willing to undergo ATI. Plasma HIV-1 RNA levels were below the lower limit of quantitation at screening and for ≥6 months before screening, and CD4^+^ T cell counts were ≥500 cells µl^−1^ at screening and at least once during the 12 months before screening. HIV-related exclusion criteria included history of AIDS-defining illness; evidence of ART initiation <3 months after acute HIV-1 infection, CD4^+^ T cell count nadir <200 cells µl^−1^ and resistance to ≥2 classes of ART or current ART regimen includes a non-nucleoside reverse transcriptase inhibitor, maraviroc or long-acting ART. Other exclusion criteria included active chronic hepatitis B or C; active, suspected or history (past 5 y) of malignancy; active, suspected or history (past 5 y) of tuberculosis; or active or history of autoimmune disease requiring systemic treatment, including adrenal insufficiency, hypo-/hyperthyroidism and autoimmune thyroiditis.

Participants were assigned a computer-generated randomization number to encode treatment group assignments according to the randomization schedule generated by study statisticians and used by a designated, unblinded site pharmacist. The site investigator and other study site personnel and the participants remained blinded throughout the study.

### Safety assessments

Safety was assessed by vital signs, physical examination, laboratory tests (including hematology and metabolic panels, thyroid function, hemolysis, plasma HIV-1 RNA CD4^+^ T cell counts) and monitoring of AEs, which were coded using the Medical Dictionary for Regulatory Activities (MedDRA, version 25.1). Investigators assessed whether an AE had a reasonable possibility of a relationship to the study drug. AESIs were immune-, infusion- or hepatic-related AEs and retroviral rebound syndrome. Virologic failures were also evaluated. All AEs were followed until satisfactory conclusion, and all suspected unexpected serious adverse reactions were reported following global and local requirements.

### Pharmacokinetics

In stage I, serial blood samples for measurement of serum budigalimab concentration were collected before administration of dose 1 (0 h) and at ≤15 min, 2 h, 4 h and 24 h after dosing on day 1; a single sample was also collected on week 1 and 2. For dose 2 at week 4, samples were collected before dosing and at ≤15 min and 2 h after dosing. Additional samples were collected at weeks 5, 6, 8, 12, 16 and 28. The same timing was used in stage II as in stage I for the collection of serial blood samples with dose 1 and follow-on doses. A single sample was collected on weeks 1, 3, 5, 7, 8, 10 and 12 and every 4 weeks until week 36. The lower limit of quantitation (LLOQ) for budigalimab was established at 27.8 ng ml^−1^.

Serum budigalimab concentrations were quantified using a validated bioanalytical assay. Pharmacokinetic parameters were calculated using noncompartmental analysis with the software Phoenix WinNonlin Version 8.0 (Certara L.P., Pharsight). The presence of ADA was determined using a validated immunoassay method. Immunogenicity of budigalimab was assessed by ADA incidence rates summarized by treatment arm. Pharmacokinetic and ADA assays were performed at a single center (Bioanalysis AbbVie Deutschland).

### PD-1 receptor saturation

Blood samples were collected for measurement of peripheral PD-1 receptor saturation on CD4^+^ and CD8^+^ T cell subsets. In stage I, samples were collected at 0, 2 and 24 h on dosing day 1 and on week 2. With the second dose on week 4, a sample was collected at 0 h. In stage II, samples were collected at 0 h on all four dosing days. Additional samples during ATI and ART restart period were collected biweekly until viral rebound, weekly or biweekly during viral rebound and every 4 weeks thereafter for ~24 weeks. Receptor saturation levels were assessed using flow cytometry. PD-1 receptor saturation assay was previously described^52^. A custom assay assessed PD-1 receptor saturation on memory T cell subtypes using a panel consisting of CD28-FITC (clone CD28.2, BD Biosciences, Catalog no. 555728), CD279-PE (clone EH.12.1, BD Biosciences, Catalog no. 560795), CD3-PERCP (clone SK7, BD Biosciences, Catalog no. 347344), CD95-BV421 (clone DX2, BD Biosciences, Catalog no. 562616), CD8-BV510 (clone SK1, BD Biosciences, Catalog no. 563919), CD4-BV605 (clone RPA-T4, BioLegend, Catalog no. 300556), isotype control-AF647 (AbbVie) and OX40-AF647 (AbbVie). An example of the gating strategy is shown in Supplementary Fig. 2a. This validated assay was performed at a single center (LabCorp). Nearly complete receptor saturation was defined as >95% PD-1 receptor saturation and was measured as the number of days after baseline [%PD-1 + (CD3^+^ /CD8^+^ cells)]/baseline [%PD-1 + (CD3^+^ /CD8^+^ cells)] was <0.05.

### ATI and viral load kinetics

In stage I and II, blood sampling for the measurement of plasma HIV-1 RNA was performed on every study visit day. Plasma HIV-1 RNA was measured using the Roche COBAS AmpliPrep/COBAS TaqMan HIV-1 Test, version 2.0 HIV-1 Assay (Roche Diagnostics). The LLOQ was 20 copies ml^−1^. HIV-1 genotypic resistance testing was performed on repeat samples during viral rebound during ATI. HIV-1 RNA assays were performed at a single center (LabCorp). Plasma concentrations of ART were determined using validated liquid chromatography with tandem mass spectrometry methods. Established LLOQ included the following: abacavir and lamivudine, 2.5 ng ml^−1^; cobicistat, 10 ng ml^−1^; bictegravir, 50 ng ml^−1^; emtricitabine, 20 ng ml^−1^; tenofovir, 5 ng ml^−1^; and dolutegravir, 20 ng ml^−1^. ART assays were performed at a single center (PPD Laboratory Services).

### HLA typing

Whole-blood genomic DNA was extracted with the QIAamp DNA Blood Mini Kit (Qiagen). Library preparation and enrichment was done using AlloSeq Tx 17 (CareDx), a next-generation sequencing-based platform that targets HLA-A, HLA-B and HLA-C and 14 other loci. The enriched libraries were loaded onto the Illumina MiSeq System and sequenced. Data analysis and allele assignments were done using the AlloSeq Assign software v1.0.2 (CareDx).

### Flow cytometry analyses of T cell subsets and phenotypes

T cell activation and proliferation analyses were carried out on CD4^+^ and CD8^+^ T cell subsets on freshly obtained anticoagulated blood samples. In the first 4 weeks of stage I of the study, samples were collected on day 1 (baseline), day 8, day 15 and day 29, with predose collections on the day of dosing. During ATI in both stages I and II, blood samples were collected before initiation of ATI and at 8- to 15-day intervals through day 57 in stage I and approximately day 85 in stage II. T cell activation and proliferation was measured at LabCorp using a validated assay that included three panels. Panel 1 included CD45RO-FITC (clone UCHL1, BD Biosciences, Catalog no. 555492), Granzyme B-PE (clone GB11, BD Biosciences, Catalog no. 561142), HLA-DR-PERCP (clone L243, BD Biosciences, Catalog no. 347364), antigen Kiel 67 (Ki67)-AF647 (clone Ki67, BioLegend, Catalog no.350510), CD4-AF700 (clone RPA-T4, BD Biosciences, Catalog no. 557922), CCR7-BV421 (clone G043H7, BioLegend, Catalog no. 353208), CD8-V500 (clone SK1, BD Biosciences, Catalog no. 561617), and CD3-BV605 (clone UCHT1, BioLegend, Catalog no. 300460); panel 2 included CD45RO, CD25-PE (clone MA251, BD Biosciences, Catalog no. 555432), HLA-DR, Ki67, CD4, CD127-BV421 (clone HIL-7R-M21, BD Biosciences, Catalog no. 562436), CD8, and CD3; and panel 3 included CD3, CD4, CD8, and isotype controls (MsIgG2a-PERCP, clone X39, BD Biosciences, Catalog no. 349054; MsIgG1-AF647, clone MOPC-21, BioLegend, Catalog no. 400130; MsIgG2a-BV421, clone MOPC-173, BioLegend, Catalog No. 400260). An example of the gating strategy is shown in Supplementary Fig. 2b. T, B, and natural killer (NK) cells were quantified on freshly obtained anticoagulated blood samples at LabCorp. The panel included CD3, CD4, CD8, CD19, CD16, and CD56 (Multitest 6-color TBNK reagent, BD Biosciences, Catalog no. 337166) to identify T, B and NK cells and CD4:CD8 ratio. An example of the gating strategy is shown in Supplementary Fig. 2c.

Peripheral blood mononuclear cells (PBMCs) were isolated from anticoagulated blood by Labcorp (Indianapolis, IN, USA). Approximate 1 million PBMCs from day 1 and during rebound in ATI period were assessed by flow cytometry to identify T cell subsets helper T cell (Th) 1, Th17, TFH-like, and CD8^+^ CXCR5^+^ cells. The panel included CD45RO-BUV395 (clone UCHL1, BD Biosciences, Catalog no.564291), CD183-BUV805 (clone 1C6, BD Biosciences, Cat# 742048), CD185-BV421 (clone RF8B2, BD Biosciences, Catalog no.562747), live/dead-BV510 (Invitrogen, Waltham, MA, USA, Cat# L34966), CD28-BV605 (clone CD28.2, BD Biosciences, Catalog no.562976), CCR7-BV650 (clone G043H7, BioLegend, Catalog no. 353234), CD161-BV711 (clone DX12, BD Biosciences, Catalog no. 563865), CD3-FITC (clone SK7, BioLegend, Catalog no. 344804), CD4-PERCP5.5 (clone RPA-T4, BioLegend, Catalog no. 300530), PD-1-PE (clone EH12.17, BioLegend, Catalog no. 329906), CD194-PECF594 (clone IG1, BD Biosciences, Catalog no. 565391), CD196-APC (clone 11A9, BD Biosciences, Catalog no. 560619), CD8-Alexa700 (clone SK1, BioLegend, Catalog no. 344724). Data were acquired with CytoFLEX LX Flow Cytometer with CytExpert Software, Beckman Coulter Life Sciences. All analyses were conducted using OMIQ software from Dotmatics. An example of the gating strategy is shown in Supplementary Fig. 2d.

HIV-specific T cell response was assessed ex-vivo through stimulation of one-million PBMCs from baseline and at the time of rebound with 1 µg peptide ml^−1^ of HIV gag peptide pool (PepMix HIV (GAG) Ultra, JPT Innovative Peptide Solutions, Catalog no. PM-HIV-GAG) with CD107a-BV421 degranulation marker (clone H4A3, BioLegend, Catalog no. 328626). After one hour incubation at 37 *°*C in 5% CO_2_ incubator, brefeldin A (BioLegend, Catalog no. 420601) and monensin (BioLegend, Catalog no. 420701) were added to the wells. Cells were stained with the following after 5 hours of incubation: live/dead-BV510 (Invitrogen, Catalog no. L34966), CD3-FITC (clone SK7, BioLegend, Catalog no. 344804), CD4-PERCP5.5 (clone RPA-T4, BioLegend, Catalog no. 300530), CD8-Alexa700 (clone SK1, BioLegend, Catalog no. 344724), CD28-APC-Cy7 (clone CD28.2, BioLegend, Catalog no. 302966), CD45RO-BV711 (clone UCHL1, BioLegend, Catalog no. 304236) and intracellularly stained with IFN-γ-PECy7 (clone 4S.B3, BioLegend, Catalog no. 502528), TNFα-BV605 (Clone Mab11, BioLegend, Catalog no. 502936) and IL-2-PE (clone MQ1-17H12, BioLegend, Catalog no. 500307). Data were acquired with CytoFLEX LX Flow Cytometer with CytExpert Software, Beckman Coulter Life Sciences. All analyses were conducted using OMIQ software from Dotmatics. An example of the gating strategy is shown in Supplementary Fig. 2e.

### Statistical methods

No power calculations for sample size considerations have been performed for this study that employed descriptive and exploratory analyses and had no planned hypothesis testing. No analysis by sex was undertaken for similar reasons. All data for participants randomized to receive budigalimab (*n* = 31) were included in the pharmacokinetic summary and parameter calculations and in the safety summaries and analyses. An ANCOVA was conducted to assess the log-transformed, dose-normalized *C*_max_ and area under the curve values and dose proportionality between the 2 mg and 10 mg IV dose of budigalimab (class variable). A repeated measures analysis was performed to assess budigalimab accumulation following the last dose relative to the first dose. Statistical analyses were performed using SAS (SAS Institute).

### Eligibility criteria

The following eligibility criteria had to be met. (1) Participants had to voluntarily sign and date an informed consent, approved by an independent ethics committee/institutional review board, before initiation of any screening or study-specific procedures.

Demographic and physical exam, laboratory, ECG/chest and X-ray assessments included the following: (2) male or female between 18 and 65 years of age inclusive at the time of screening; (3) body mass index ≥18.0 to <35.0 kg/m^2^ after rounding to the tenth decimal (calculated as weight in kilograms divided by the square of height measured in meters) and (4) weight of at least 35 kg; (5) condition of general good health based upon the results of a medical history, physical examination, vital signs, laboratory profile and a 12-lead ECG; and (6) no clinically significant laboratory values at screening that would pose undue risk for the individual or interfere with safety assessments (per the investigator). (7) Participants needed to have laboratory values that met the following criteria at screening: (a) Absence of current HBV infection on screening tests, defined as a positive HBsAg, or HBV DNA > LLOQ in those with isolated positive anti-HBc (that is, negative HbsAg and anti-HBs). (b) Absence of detectable HCV RNA result if HCV antibody positive. Participants with a history of hepatitis C who achieved SVR12 after anti-viral therapy may be enrolled if all eligibility criteria are met: hemoglobin A1C level ≤ 7%; TSH and T4 results within reference range; negative anti-thyroglobulin antibody and anti-thyroid peroxidase antibody; ACTH results within reference range; ANC ≥ 1,000 μl^−1^; hemoglobin ≥ LLN; calculated creatinine clearance (using Cockcroft-Gault method) of ≥ 60 ml min^−1^; platelets ≥ 125,000 cells per mm^3^; ALT and AST ≤ 1.25× upper limit of normal (ULN); total bilirubin ≤ 1.1 × ULN (unless taking atazanavir or known history of Gilbert’s syndrome); INR ≤ 1.1 × ULN. (c) Negative screen for drugs of abuse and alcohol at screening was required. Patients with a positive marijuana screen could be included after evaluation by the investigator that the use would not interfere with adherence to study requirements. (8) No clinically significant abnormality on screening chest X-ray, including calcified granuloma and/or pleural scarring. (9) Absence of clinically significant abnormality detected on ECG regarding rate, rhythm or conduction (for example, QT interval corrected for heart rate using Fridericia’s formula should be <450 ms for males and < 460 ms for females). Regarding disease (HIV) activity, the following HIV-specific laboratory parameters must be met at screening: positive test result for anti-HIV antibody at screening; CD4 cell count ≥500 cells μl^−1^ at screening and at least once during the 12 months before screening; plasma HIV-1 RNA LLOQ at screening and for at least 6 months before screening (by an approved plasma HIV-1 RNA quantitative assay including but not limited to: COBAS Ampliprep/COBAS Taqman HIV-1 Test, v 2.0 or Abbott RealTime HIV-1 assay); a single detectable HIV-1 RNA but less than 200 copies ml^−1^ before screening was allowed if followed by HIV-1 RNA below quantifiable limits; and negative HIV-2 Ab at screening. (11) On ART for at least 12 months before screening and on current ART regimen for at least 8 weeks before screening (regimen cannot include a non-nucleoside reverse transcriptase inhibitor, maraviroc, or long-acting ART regimens. (12) Ability and willingness to remain on ART throughout the study (except during the ART interruption period). (13) Must be willing to undergo ART interruption and change ART regimen if required. (14) No history of AIDS-defining illness. (15) No known evidence of initiation of ART <3 months after acute HIV-1 infection (for example, initiation of ART based on a positive HIV-1 Ab obtained 1 month after previously negative HIV-1 Ab, or initiation of ART based on a positive HIV-1 RNA and/or p24 antigen but negative HIV-1 Ab (Fiebig I-II)). (16) No known resistance to ≥2 classes of ART. (17) History of CD4^+^ T cell nadir ≥200 cells μl^−1^ during chronic stage of infection. If documentation for these criteria on HIV history is not available, subject recall may also be used for evaluation. (18) No history of or active immunodeficiency (other than HIV). (19) No active autoimmune disease or history of autoimmune disease that has required systemic treatment (that is, with use of disease-modifying agents, corticosteroids, or immunosuppressive drugs) including but not limited to inflammatory bowel diseases, scleroderma, severe psoriasis, myocarditis, uveitis, pneumonitis, systemic lupus erythematosus, rheumatoid arthritis, optic neuritis, myasthenia gravis, adrenal insufficiency, hypothyroidism and/or hyperthyroidism, autoimmune thyroiditis, hypophysitis or sarcoidosis. (20) Subject must not have had prior therapy/exposure to ABBV-181 or any other immune checkpoint inhibitor (for example, anti-PD-1, anti-PD-L1, anti-PD-L2, anti-CTLA4). (21) Subject must not have had prior or current treatment with immunoglobulin therapy. (22) No clinically significant medical disorders (other than HIV-1 infection) that might expose the subject to undue risk of harm, confound study outcomes or prevent the subject from completing the study, including but not limited to significant or unstable cardiac disease (for example, congestive heart failure, angina, myocardial infarction), significant pulmonary disease (for example, chronic obstructive pulmonary disease, asthma requiring systemic therapy), significant neurologic disease (for example, stroke, dementia), chronic active infectious disease (except for HIV) (for example, sinusitis, chronic chest infection), chronic liver disease, poorly controlled diabetes mellitus or history of Stevens-Johnson syndrome, toxic epidermal necrolysis or drug reaction with eosinophilia and systemic symptoms. (23) No history of any clinically significant illness/infection/major febrile illness, hospitalization, or any surgical procedure within 30 days prior to the first dose of study drug. (24) No evidence of ongoing hemolysis on screening hemolysis panel (total, direct and unconjugated serum bilirubin, LDH, peripheral blood smear, D-dimer and serum haptoglobin) as judged by the investigator. (25) No active or suspected malignancy or history of malignancy (other than basal cell skin cancer or cervical carcinoma in situ) in the past 5 years. (26) No history of or active tuberculosis at screening. (27) No history of positive tuberculosis skin test or interferon gamma release assay (IGRA) that is considered clinically significant by the investigator. Must have a negative result for an IGRA at screening unless subject had a documented negative IGRA result within 90 days before screening and nothing has changed in subject’s medical history to warrant a repeat test at screening. (28) No history of a severe, life-threatening, or significant sensitivity to any excipients of the study drug. (29) No history of major immunologic reaction to any IgG-containing agent. (30) Has not donated blood (including plasmapheresis), lost ≥550 ml blood volume, or received a transfusion of any blood product within 8 weeks before study drug administration. (31) No known psychiatric or substance abuse disorders that would interfere with adherence to study requirements. (32) Is not currently enrolled in another interventional clinical study. (33) Has not been previously enrolled in this study. (34) In the opinion of the investigator, this individual is a suitable candidate for enrollment in the study. (35) Contraception and prevention of HIV transmission, including a negative serum pregnancy test result at screening and negative urine pregnancy test result on the study day 1 visit before the first dose of study drug (females of childbearing potential only). (36) Female subjects of childbearing potential must agree to use an effective barrier method of protection during sexual activity from study day 1 through last study visit for the purposes of prevention of HIV transmission. In addition, female subjects of childbearing potential must also use an additional protocol-specified effective method of contraception for the purposes of birth control from study day 1 through last study visit (including for at least 6 months after the last dose of study drug for subjects who discontinue the study early). (37) Female subjects of non-childbearing potential must agree to use an effective barrier method of protection during sexual activity from study day 1 through last study visit to prevent HIV transmission. Non-childbearing potential is defined as postmenopausal, age >55 years with no menses for 12 or more months without an alternative medical cause; postmenopausal, age ≤55 years with no menses for 12 or more months without an alternative medical cause and a follicle-stimulating hormone level > 40 IU liter^−1^; permanently surgically sterile (bilateral oophorectomy, bilateral salpingectomy or hysterectomy). (38) Female who is not pregnant or breastfeeding and is not considering becoming pregnant or donating eggs during the study. (39) Male subjects must agree to use an effective barrier method of protection during sexual activity from study day 1 through last study visit (including for at least 6 months after the last dose of study drug for subjects who discontinue the study early) for the purposes of both contraception and prevention of HIV transmission. (40) Male who is not considering fathering a child or donating sperm during the study. (41) Subject must not have received immunomodulatory (for example, interleukins, interferons, tumor necrosis factor modifiers) or immunosuppressive (including intravenous or oral steroids at any dose, but excluding steroids that are inhaled, topical or via local injection) therapy within 24 weeks before the first dose of study drug. (42) Subjects must not have been treated with any investigational drug within 30 days or 5 half-lives of the drug (whichever is longer) prior to the first dose of study drug. (43) Subject must not have received any live vaccine within 4 weeks before the first dose of study drug. (44) Subject must not have signs/symptoms associated with COVID-19. (45) Absence of newly confirmed, acute SARS-CoV-2 infection by a PCR test completed within 7 days before day 1 dose.

### Reporting summary

Further information on research design is available in the Nature Portfolio Reporting Summary linked to this article.

## Online content

Any methods, additional references, Nature Portfolio reporting summaries, source data, extended data, supplementary information, acknowledgements, peer review information; details of author contributions and competing interests; and statements of data and code availability are available at 10.1038/s41591-025-03993-0.

## Supplementary information

Supplementary Information Supplementary Figures 1 and 2.

Reporting Summary

## Figures and Tables

**Extended Data Table 1:**
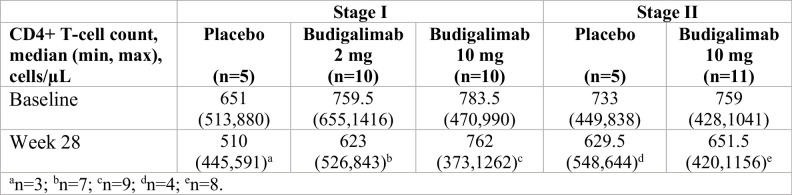
Median CD4 + T cell counts over time across all study arms

**Extended Data Table 2:**
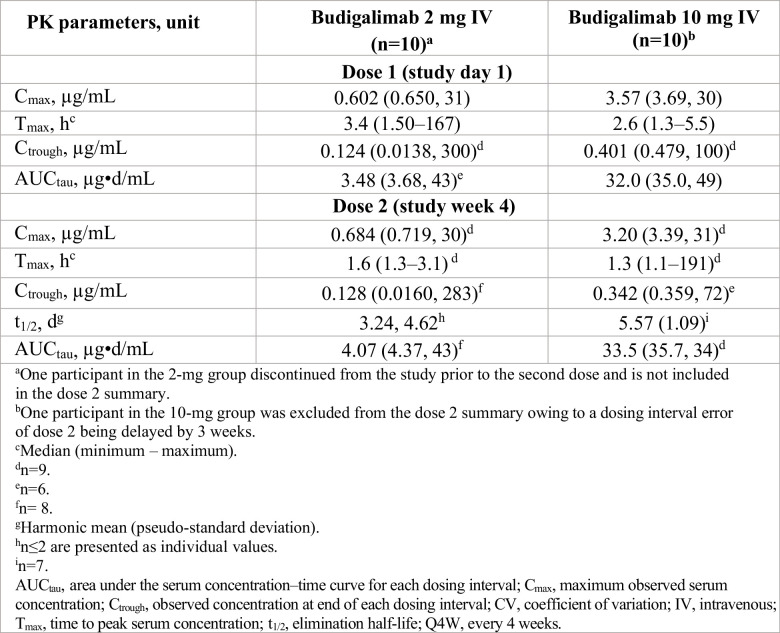
Geometric mean (mean, %CV) budigalimab pharmacokinetic parameters following Q4W administration in stage I

**Extended Data Table 3:**
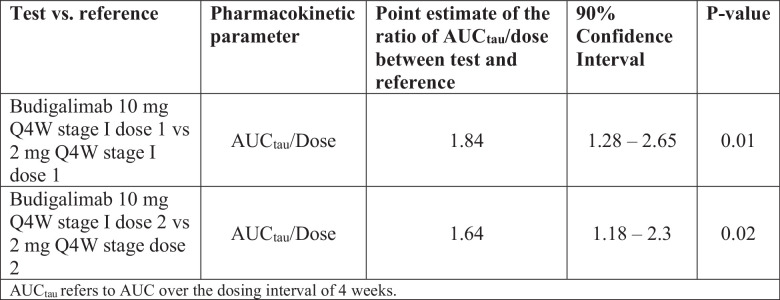
Analysis of covariance of the dose-normalized area under the serum concentration–time curve over the dosing interval (AUCtau) following 2 mg and 10 mg Q4W dosing of budigalimab in Stage I

**Extended Data Table 4:**
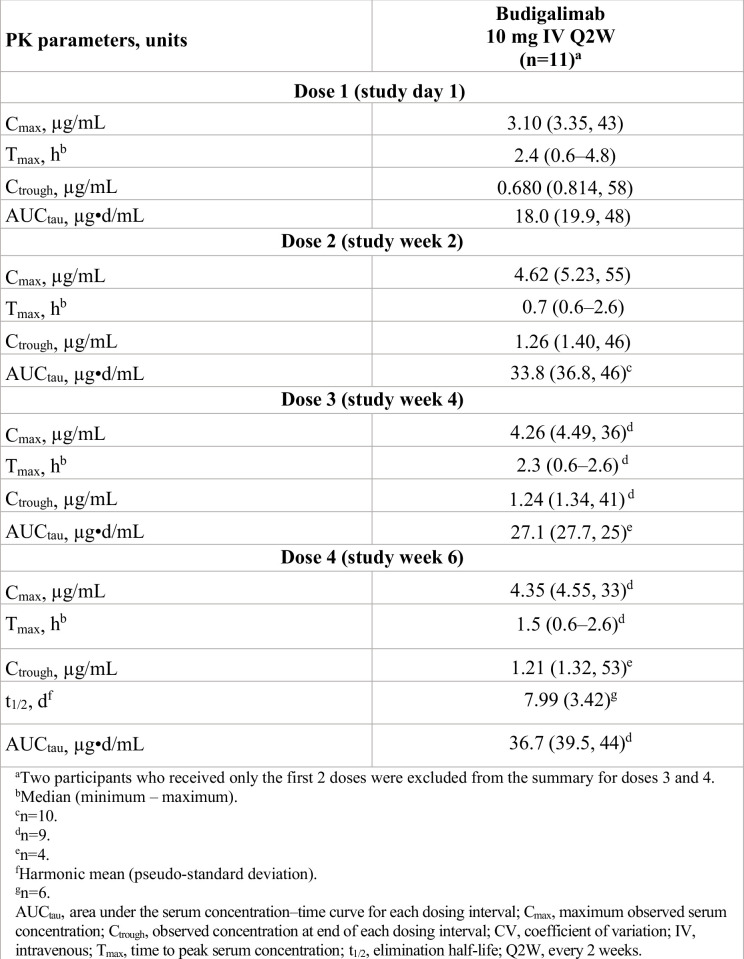
Geometric mean (mean, % CV) budigalimab pharmacokinetic parameters following Q2W administration in stage II

**Extended Data Table 5:**
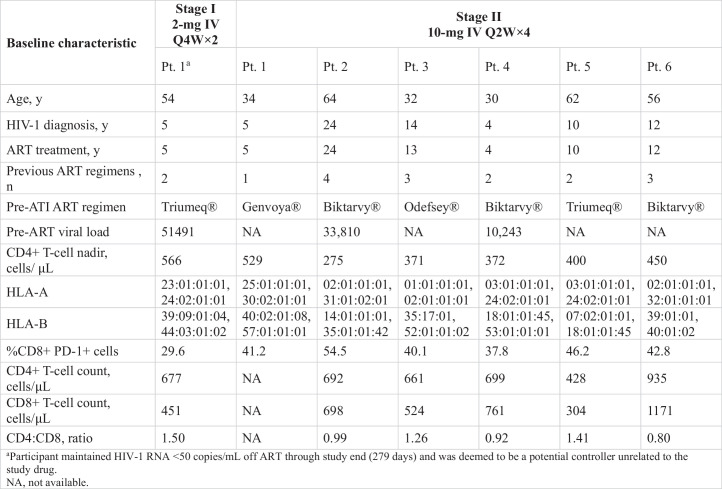
Baseline characteristics of individual participants who did not experience a rebound or experienced a delayed viral rebound with a low viral load and/or viral load resuppression ( < 1000 cp/ml) after rebound

**Extended Data Table 6:**
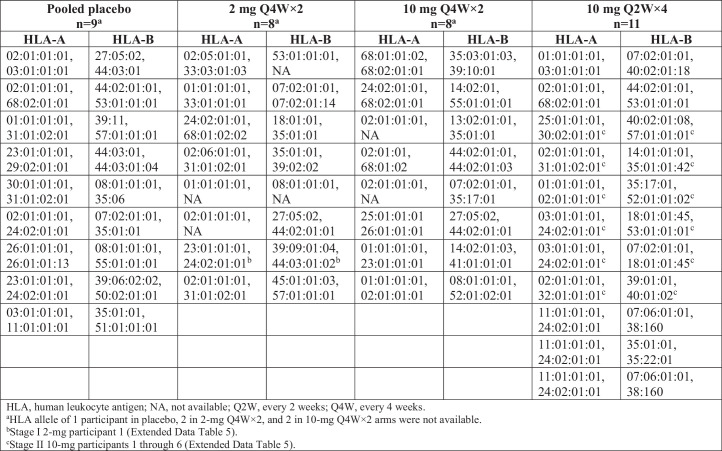
HLA alleles

## Data Availability

AbbVie is committed to responsible data sharing regarding the clinical trials we sponsor. This includes access to anonymized, individual, and trial-level data (analysis datasets), as well as other information (eg, protocols, clinical study reports, or analysis plans), as long as the trials are not part of an ongoing or planned regulatory submission. This includes requests for clinical trial data for unlicensed products and indications. These clinical trial data can be requested by any qualified researchers who engage in rigorous, independent, scientific research and will be provided following review and approval of a research proposal, statistical analysis plan (SAP), and execution of a data sharing agreement (DSA). Data requests can be submitted at any time after approval in the US and Europe and after acceptance of this manuscript for publication. The data will be accessible for 12 months, with possible extensions considered. For more information on the process or to submit a request, visit the following https://vivli.org/ourmember/abbvie/ then select “Home”.
