# Supplementary material for: Budigalimab, an anti-PD-1 inhibitor, for people living with HIV-1: a randomized, placebo-controlled phase 1b study
Source: Nat Med. 2025 Oct 15;31(11):3879–88. doi: 10.1038/s41591-025-03993-0 (PMC12618229; doi:10.1038/s41591-025-03993-0)

nature medicine

Supplementary information https://doi.org/10.1038/s41591-025-03993-0

Budigalimab, an anti-PD-1 inhibitor, for people living with HIV-1: a randomized, placebo-controlled phase 1b study

In the format provided by the authors and unedited

SUPPLEMENTARY MATERIALS

Budigalimab, an anti-PD-1 inhibitor, for people living with HIV-1: a randomized, placebo-controlled phase 1b study

Table of Contents

Supplementary Fig. 1 2

Supplementary Fig. 2 3

Supplementary Fig. 1 HIV specific T cell responses to HIV gag peptide pool. HIV-specific T-cell responses were evaluated by stimulating PBMCs collected at baseline and during ATI with HIV gag and/or pol peptide pools by intracellular cytokine staining assay. T-cell responses were measured as the frequency of CD4+ and CD8+ T cells expressing degranulation marker CD107a or cytokine markers IFN-γ, TNF-α, and IL-2. In this limited analysis, T-cell responses were predominantly observed on CD8+ T-cell population; gag peptide pool responses were more pronounced than responses to pol peptide pool. Percentage of CD8+ T cells secreting cytokines IFN-γ, TNF-α, and degranulation marker CD107a in available samples are shown in figures A-C and fold changes in figures D-F. Frequency of cells secreting IL-2 was negligible and are not shown.

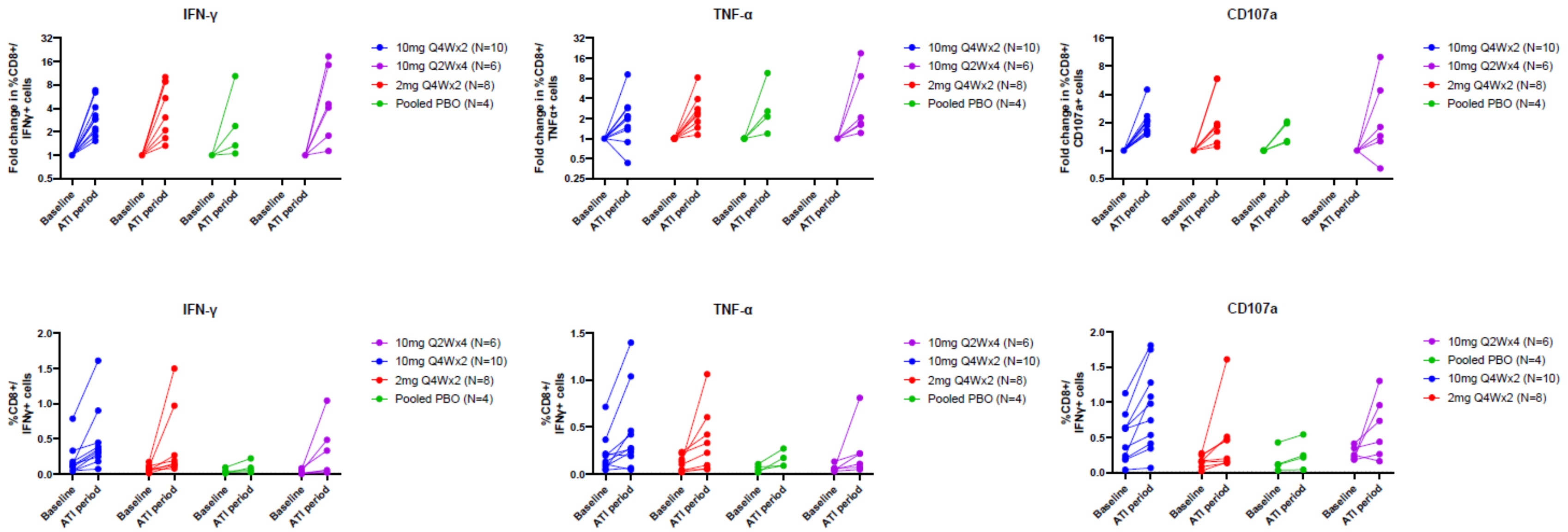

Supplementary Fig. 2 Example gating strategy for flow cytometry-based analyses. (A) Gating strategy for PD-1 expression on CD8+ T cells; (B) Gating strategy for T cell proliferation and activation markers (Ki67, HLA-DR and Granzyme B); (C) Gating strategy for TBNK cells phenotyping; (D) Gating strategy for T cell subsets T follicular helper-like (TFH) cells, peripheral CXCR5+CD8+ T cells, and CCR6+CD4+ T cells; (E) Gating strategy for intracellular cytokine staining assay measuring degranulation marker CD107a and cytokine markers IFN-γ and TNF-α.

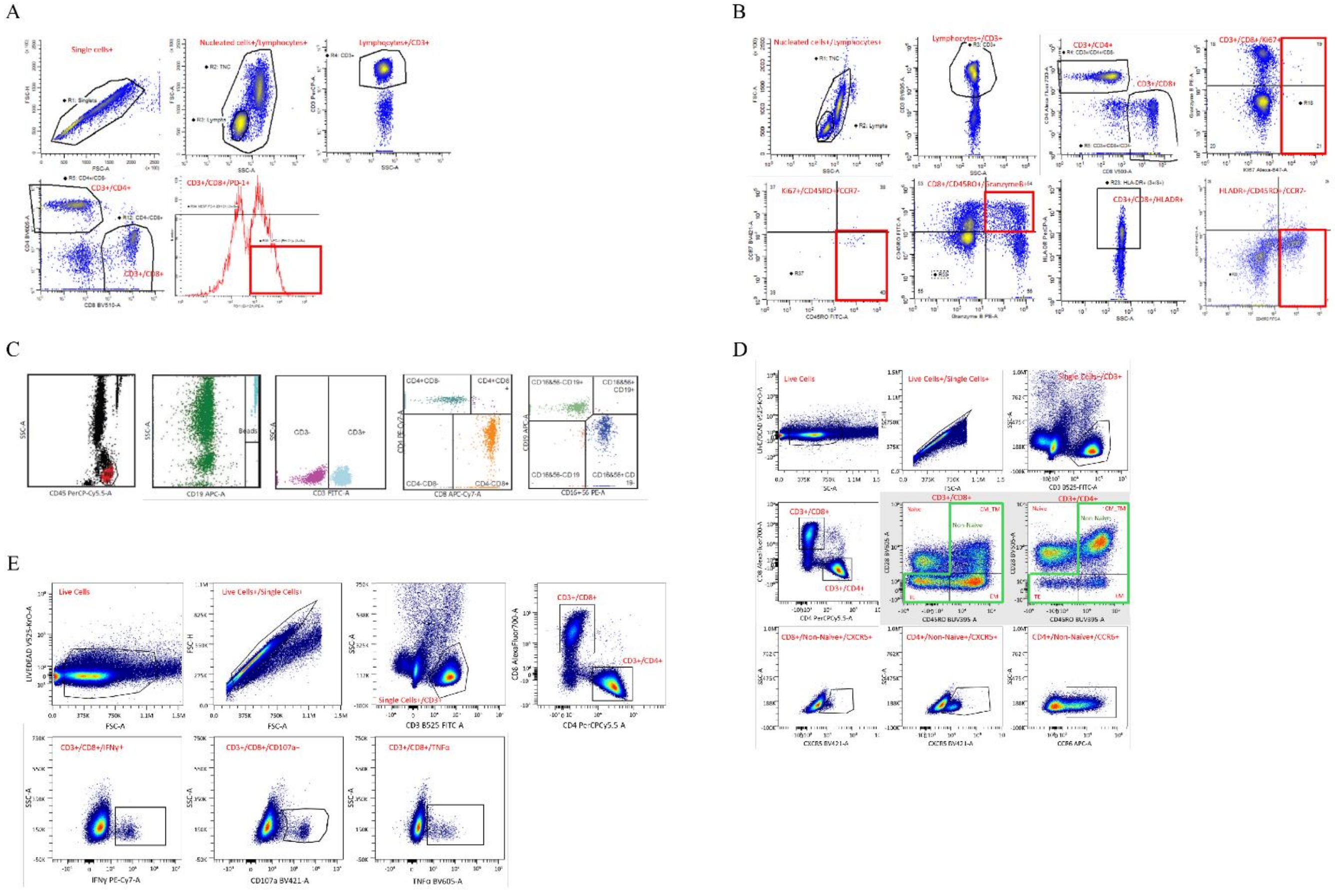

Supplement: Supplementary file 1 — Supplementary Figures 1 and 2. [file 41591_2025_3993_MOESM1_ESM.pdf]
