# Supplementary material for: Budigalimab, an anti-PD-1 inhibitor, for people living with HIV-1: a randomized, placebo-controlled phase 1b study
Source: Nat Med. 2025 Oct 15;31(11):3879–88. doi: 10.1038/s41591-025-03993-0 (PMC12618229; doi:10.1038/s41591-025-03993-0)
Supplement: Supplementary file 2 — Reporting Summary [file 41591_2025_3993_MOESM2_ESM.pdf]

nature portfolio

Corresponding author(s): Jean-Pierre Routy

Last updated by author(s): Aug 15, 2025

Reporting Summary

Nature Portfolio wishes to improve the reproducibility of the work that we publish. This form provides structure for consistency and transparency in reporting. For further information on Nature Portfolio policies, see our Editorial Policies and the Editorial Policy Checklist.

Statistics

For all statistical analyses, confirm that the following items are present in the figure legend, table legend, main text, or Methods section.

n/a	Confirmed	
☐	☒	The exact sample size (*n*) for each experimental group/condition, given as a discrete number and unit of measurement
☐	☒	A statement on whether measurements were taken from distinct samples or whether the same sample was measured repeatedly
☒	☐	The statistical test(s) used AND whether they are one- or two-sided<br>*Only common tests should be described solely by name; describe more complex techniques in the Methods section.*
☒	☐	A description of all covariates tested
☒	☐	A description of any assumptions or corrections, such as tests of normality and adjustment for multiple comparisons
☐	☒	A full description of the statistical parameters including central tendency (e.g. means) or other basic estimates (e.g. regression coefficient) AND variation (e.g. standard deviation) or associated estimates of uncertainty (e.g. confidence intervals)
☒	☐	For null hypothesis testing, the test statistic (e.g. *F*, *t*, *r*) with confidence intervals, effect sizes, degrees of freedom and *P* value noted<br>*Give P values as exact values whenever suitable.*
☒	☐	For Bayesian analysis, information on the choice of priors and Markov chain Monte Carlo settings
☒	☐	For hierarchical and complex designs, identification of the appropriate level for tests and full reporting of outcomes
☒	☐	Estimates of effect sizes (e.g. Cohen's *d*, Pearson's *r*), indicating how they were calculated

Our web collection on statistics for biologists contains articles on many of the points above.

Software and code

Policy information about availability of computer code

Data collection	This study used the Medidata Rave Electronic Data Capture System for data collection.
Data analysis	Statistical analyses were performed using SAS (SAS Institute, Inc., Cary, NC, USA).

For manuscripts utilizing custom algorithms or software that are central to the research but not yet described in published literature, software must be made available to editors and reviewers. We strongly encourage code deposition in a community repository (e.g. GitHub). See the Nature Portfolio guidelines for submitting code & software for further information.

Data

Policy information about availability of data

All manuscripts must include a data availability statement. This statement should provide the following information, where applicable:

- Accession codes, unique identifiers, or web links for publicly available datasets
- A description of any restrictions on data availability
- For clinical datasets or third party data, please ensure that the statement adheres to our policy

AbbVie is committed to responsible data sharing regarding the clinical trials we sponsor. This includes access to anonymized, individual, and trial-level data (analysis datasets), as well as other information (eg, protocols, clinical study reports, or analysis plans), as long as the trials are not part of an ongoing or planned regulatory submission. This includes requests for clinical trial data for unlicensed products and indications. These clinical trial data can be requested by any qualified researchers who engage in rigorous, independent, scientific research and will be provided following review and approval of a research proposal, statistical analysis

plan (SAP), and execution of a data sharing agreement (DSA). Data requests can be submitted at any time after approval in the US and Europe and after acceptance of this manuscript for publication. The data will be accessible for 12 months, with possible extensions considered. For more information on the process or to submit a request, visit the following https://vivli.org/ourmember/abbvie/
then select “Home”.

Research involving human participants, their data, or biological material

Policy information about studies with human participants or human data. See also policy information about sex, gender (identity/presentation), and sexual orientation and race, ethnicity and racism.

Reporting on sex and gender	Sex (female/male) and gender (female/male/non-binary/transgender male/transgender female/other/non-disclosure) were self-reported by the study participants.
Reporting on race, ethnicity, or other socially relevant groupings	Race and ethnicity was collected along with other demographic categories, and was self-reported by the study participants. Study participants could report more than one race. Participant demographics and baseline characteristics were balanced across treatment groups with representation of eligibility criteria, except for sex. Participant demographics are provided in Table 2.
Population characteristics	Adults living with HIV, 18-65 years old, stable on antiretroviral therapy (ART) for at least 12 months (CD4+ T cell count >= 500 cells/uL and Plasma HIV-1 RNA below lower limit of quantification (LLOQ), in general good health, with no known resistance to 2 classes of ART that were willing to undergo ART interruption.
Recruitment	Participants that meet inclusion and exclusion criteria were recruited from pre-selected sites across the US and Australia. Sites were required to have extensive experience in conducting early and complex clinical trials in people living with HIV as well as a multi-professional care team. Sites received guidance to select participants that have been in their care and were known by site staff. Recruitment started on late 2019 and went throughout COVID pandemic which may have limited participation to individuals with easier access to the study site.
Ethics oversight	Sites used a central institutional review board (Advarra, Inc., Columbia, MD) or local ethics committees to have the study protocol, informed consent, and participant information approved. The study was conducted in accordance with International Council for Harmonisation (ICH) guidelines, and applicable regulations, guidelines, and principles had their origin in the Declaration of Helsinki. Written informed consent, including a full discussion of risks of budigalimab, risks of ATI, and conditions for ART reinitiation, was obtained for each participant before screening or study-specific procedures

Note that full information on the approval of the study protocol must also be provided in the manuscript.

Field-specific reporting

Please select the one below that is the best fit for your research. If you are not sure, read the appropriate sections before making your selection.

☒ Life sciences ☐ Behavioural & social sciences ☐ Ecological, evolutionary & environmental sciences

For a reference copy of the document with all sections, see nature.com/documents/nr-reporting-summary-flat.pdf

Life sciences study design

All studies must disclose on these points even when the disclosure is negative.

Sample size	No power calculations for sample size considerations have been performed for this study that employed descriptive and exploratory analyses and had no planned hypothesis testing.
Data exclusions	None
Replication	All data presented are biological replicates.
Randomization	Participants were assigned a computer-generated randomization number to encode treatment group assignments according to the randomization schedule generated by study statisticians and used by a designated, unblinded site pharmacist. The site investigator and other study site personnel and the participants remained blinded throughout the study.
Blinding	The site investigator and other study site personnel and the participants remained blinded throughout the study.

Reporting for specific materials, systems and methods

We require information from authors about some types of materials, experimental systems and methods used in many studies. Here, indicate whether each material, system or method listed is relevant to your study. If you are not sure if a list item applies to your research, read the appropriate section before selecting a response.

Materials & experimental systems

n/a	Involved in the study
☐	☒ Antibodies
☒	☐ Eukaryotic cell lines
☒	☐ Palaeontology and archaeology
☒	☐ Animals and other organisms
☐	☒ Clinical data
☒	☐ Dual use research of concern
☒	☐ Plants

Methods

n/a	Involved in the study
☒	☐ ChIP-seq
☐	☒ Flow cytometry
☒	☐ MRI-based neuroimaging

Antibodies

Antibodies used

CD45RO-BUV395 (clone UCHL1, BD Biosciences, Franklin Lakes, New Jersey, USA, Catalog no.564291), CD183-BUV805 (clone 1C6, BD Biosciences, Cat# 742048), CD185-BV421 (clone RF8B2, BD Biosciences, Catalog no.562747), live/dead BV510 (Invitrogen, Waltham, Massachusetts, USA, Cat# L34966), CD28-BV605 (clone CD28.2, BD Biosciences, Catalog no.562976), CCR7-BV650 (clone G043H7, BioLegend, San Diego, California, USA, Catalog no. 353234), CD161-BV711 (clone DX12, BD Biosciences, Catalog no. 563865), CD3-FITC (clone SK7, BioLegend, Catalog no. 344804), CD4-PERCP5.5 (clone RPA-T4, BioLegend, Catalog no. 300530), PD-1-PE (clone EH12.17, BioLegend, Catalog no. 329906), CD194-PECF594 (clone IG1, BD Biosciences, Catalog no. 565391), CD196-APC (clone 11A9, BD Biosciences, Catalog no. 560619), CD8-Alexa700 (clone SK1, BioLegend, Catalog no. 344724), CD28-APC-Cy7 (clone CD28.2, Biolegend, Catalog no. 302966), CD45RO-BV711 (clone UCHL1, Biolegend, Catalog no. 304236), and intracellularly stained with IFNγ-PECy7 (clone 4S.B3, Biolegend, Catalog no. 502528), TNFα-BV605 (Clone Mab11, Biolegend, Catalog no. 502936) and IL-2-PE (clone MQ1-17H12, Biolegend, Catalog no. 500307), MultitestTM 6-color TBNK reagent, Catalog no. 337166, , CD28-FITC (clone CD28.2, BD Biosciences, Franklin Lakes, New Jersey, USA, Catalog no. 555728), CD279-PE (clone EH.12.1, BD Biosciences, Catalog no. 560795), CD3-PERCP (clone SK7, BD Biosciences, Catalog no. 347344), CD95-BV421 (clone DX2, BD Biosciences, Catalog no. 562616), CD8-BV510 (clone SK1, BD Biosciences, Catalog no. 563919), CD4-BV605 (clone RPA-T4, BioLegend, San Diego, California, USA, catalog no. 300556), isotype control-AF647 (AbbVie Inc., North Chicago, Illinois, USA) and OX40-AF647 (AbbVie Inc., North Chicago, Illinois, USA), CD45RO-FITC (clone UCHL1, BD Biosciences, Catalog no. 555492), Granzyme B-PE (clone GB11, BD Biosciences, Catalog no. 561142) , human leukocyte antigen (HLA)-DR-PERCP (clone L243, BD Biosciences, Catalog no. 347364), antigen Kiel 67 (Ki67)-AF647 (clone Ki-67, Biolegend, Catalog no.350510), CD4-AF700 (clone RPA-T4, BD Biosciences, Catalog no. 557922), CCR7-BV421 (clone G043H7, Biolegend, Catalog no. 353208), CD8-V500 (clone SK1, BD Biosciences, Catalog no. 561617), and CD3-BV605 (clone UCHT1, Biolegend, Catalog no. 300460), CD25-PE (clone MA251, BD Biosciences, Catalog no. 555432), CD127-BV421 (clone HIL-7R-M21, BD Biosciences, Catalog no. 562436), MsIgG2a-PERCP, clone X39, BD Biosciences, Catalog no. 349054; MsIgG1-AF647, clone MOPC-21, Biolegend, Catalog no. 400130; MsIgG2a-BV421, clone MOPC-173, Biolegend, Catalog No. 400260

Validation

1.5ug per reaction were used as per manufacturer's recommendation on antibody usage; some assays were validated and supported by Labcorp (Indianapolis, IN, USA)

Clinical data

Policy information about clinical studies

All manuscripts should comply with the ICMJE guidelines for publication of clinical research and a completed CONSORT checklist must be included with all submissions.

Clinical trial registration

NCT04223804

Study protocol

N/A

Data collection

Participants were enrolled from 11 sites in the US (9 sites), Canada (1 site), and Australia (1 site). The first participant visit occurred January 30, 2020, and the last participant visit occurred February 27, 2023.

Outcomes

The safety, pharmacokinetics, and pharmacodynamics of multiple intravenous doses of low-dose (2-10 mg) budigalimab were assessed in people living with HIV.

Plants

Seed stocks

N/A

Novel plant genotypes

N/A

Authentication

N/A

Flow Cytometry

Plots

Confirm that:

☒ The axis labels state the marker and fluorochrome used (e.g. CD4-FITC).

☒ The axis scales are clearly visible. Include numbers along axes only for bottom left plot of group (a 'group' is an analysis of identical markers).

☒ All plots are contour plots with outliers or pseudocolor plots.

☒ A numerical value for number of cells or percentage (with statistics) is provided.

Methodology

Sample preparation	Anticoagulated whole blood or peripheral blood mononuclear cells isolated from anticoagulated blood
Instrument	CytoFLEX LX Flow Cytometer; Becton Dickinson FACSCanto ▯II
Software	OMIQ software from Dotmatics; BD FACSCanto Clinical software version 2.2 or higher
Cell population abundance	Cell populations were gated on live single cells. Figures include cell frequency. An example of gating strategy is provided in Supplemental Figure 2A-E.
Gating strategy	An example of the gating strategy is provided in Supplemental Figure 2A-E.

☒ Tick this box to confirm that a figure exemplifying the gating strategy is provided in the Supplementary Information.
